# Clinical prediction models for mortality and functional outcome following ischemic stroke: A systematic review and meta-analysis

**DOI:** 10.1371/journal.pone.0185402

**Published:** 2018-01-29

**Authors:** Marion Fahey, Elise Crayton, Charles Wolfe, Abdel Douiri

**Affiliations:** Division of Health and Social Care Research, Faculty of Life Sciences and Medicine, King’s College London, London, United Kingdom; University of Glasgow, UNITED KINGDOM

## Abstract

**Objective:**

We aim to identify and critically appraise clinical prediction models of mortality and function following ischaemic stroke.

**Methods:**

Electronic databases, reference lists, citations were searched from inception to September 2015. Studies were selected for inclusion, according to pre-specified criteria and critically appraised by independent, blinded reviewers. The discrimination of the prediction models was measured by the area under the curve receiver operating characteristic curve or c-statistic in random effects meta-analysis. Heterogeneity was measured using I2. Appropriate appraisal tools and reporting guidelines were used in this review.

**Results:**

31395 references were screened, of which 109 articles were included in the review. These articles described 66 different predictive risk models. Appraisal identified poor methodological quality and a high risk of bias for most models. However, all models precede the development of reporting guidelines for prediction modelling studies. Generalisability of models could be improved, less than half of the included models have been externally validated(n = 27/66). 152 predictors of mortality and 192 predictors and functional outcome were identified. No studies assessing ability to improve patient outcome (model impact studies) were identified.

**Conclusions:**

Further external validation and model impact studies to confirm the utility of existing models in supporting decision-making is required. Existing models have much potential. Those wishing to predict stroke outcome are advised to build on previous work, to update and adapt validated models to their specific contexts opposed to designing new ones.

## Introduction

Although stroke incidence, prevalence, mortality and disability-adjusted-life-year rates have declined over the last 20 years, the overall burden of stroke in terms of absolute number of people affected by, or who remain disabled from, stroke has increased across the globe in both men and women of all ages[1].

Prediction models (pms), which combine patient characteristics and care processes to estimate the probability of developing a particular event or outcome in the future (prognosis), have proven valuable in the primary prevention of Stroke. Pms such as the Framingham Score (2008)[2], QRISK (2007)[3], Reynolds men(2008) [4], Reynolds women (2007)[5], and EURO-SCORE(2003) [6] have been used in stroke to inform health service planning and stratified care, to support clinical decision making, diagnostic work up and choice of therapy in high risk groups [7] and to identify enrichment samples in clinical trials [7].

The most important goal for stroke is a reduction in incidence of future events—prevention is always better than cure. However the need for rehabilitation and long term follow up efforts to improve functional outcome and prevent mortality should also be recognized as important measures to sustainably reduce the burden of stroke[1]. As over sixty nine per cent of those who experience a stroke are dependant (Barthel index <20) adequate attention should be paid to secondary stroke prevention.

Although it has been suggested that the PMs are also valuable in tertiary prevention of stroke, implementation is poor, even where models are robust[8]. Application may be limited by inadequate statistical performance of pmss, particularly with respect external populations, concerns over usability and reliability and failure to assess clinical impact[9].

The aim of this systematic review and meta-analysis is to identify pms for survival and functional outcome[10] following stroke and to appraise these models using current guidelines and to determine the pooled accuracy of identified models[7–9,11–15]. We have chosen to focus on Ischemic stroke outcomes as approximately 80% of stroke are ischemic subtype.

## Materials and methods

We followed the Critical Appraisal and Data Extraction for Systematic Reviews of Prediction Modelling Studies (CHARMS) Checklist to guide the framing of the review aim, search strategy, and study inclusion and exclusion criteria (S1 and S5 Tables)[14].For details of the review protocol, criteria for eligibility and search methods (S1 Text, S1 Table and S3 Text). This review was completed consistent with PRISMA guidelines (S4 Table) [16].

### Search strategy

Models for review were identified by searching: MEDLINE, EMBASE, CINHAL and the Cochrane Database of Systematic Reviews (CDSR) from inception to September 2015, all publications by the Cochrane Prognosis Methods Research Group (PMG) and searching the reference lists and citations of included studies.The search strategy (S3 Text, S2 and S3 Tables) comprised a combination of key word and free text searching and incorporated a validated prognostic research search filter (sensitivity: 0·98[0·92, 1·00]; specificity: 0·86 [0·85, 0·87])[17],[18]. The Cochrane Groups search filter for stroke was initially incorportated into the search strategy but later replaced with ‘stroke’as a keyword becuase it was not feasible to screen the large number of returned studies. This is considred justified as reference list and citation searching was also employed.

### Selection criteria

Populations for this review and meta-analysis were broadly inclusive, involving any country, both sexes and patients managed in the community or in hospital. The target population are individuals who have had an ischemic stroke, as defined by the included study’s authors. Models predicting outcome following pediatric stroke, recurrent stroke or specific patient subgroups (i.e. Patients with comorbidities) which do not reflect the general ischemic stroke population were excluded. Models which do not distinguish between stroke type, but are suitable for ischemic stroke risk prediction, were included regardless of whether ischemic stroke data could be differentiated or not. This is justified as we are interested to synthesis all models which predict outcome from ischemic stroke.

The end points of interest were mortality and functional outcome and the start point time of stroke. Studies examining both mortality and function, as a composite outcome i.e. ‘alive and independent’ were also included. In order to capture PM predicting function and mortality in the short and long term, a timeframe for outcome measurement was not specified. This review measures functional outcome in the domain of activities [10]. Salter and colleagues have identified specific outcome measurement tools, which fall under this definition[19,20]. The domain Body structure and functions has not been considered as these models typically predict recovery of as specific limb opposed to a global disbility measure. The Particpation domain has also been omitted as there is debate regarding the most important indicators of successful involvement in a life situation and which ones best represent the societal perspective of functioning.[21]

Five distinct approaches to multivariable prediction research have been identified [22].Prognostic factor studies are excluded from this review due to the high risk of publication bias and false positive studies [23, 24].To be eligible for inclusion model development studies must have a minimum of ten events per variable considered in the model and validation studies must have a minimum of 200 events and 200 non events, as this was a previously accepted convention [25–27].

### Data extraction, assessment and synthesis

Studies were selected for inclusion, data extracted and appraised in duplicate by independent review author (MF, EC, AM). Disagreement was resolved by discussion or independently by a third review author as arbiter (AD). CHARMS Checklist was used for appraisal and the Prediction Study Risk of Bias Tool (PROBAST) was used to assess bias [14, 15]. PROBAST is being piloted and is likely to be published in the near future.

### Statistical analysis

Validation studies were aggregated by model and by outcome. Models or outcomes for which there were five or more studies reporting accuracy were included in the meta-analysis. Given the small number of eligible studies and generally poor quality, studies were included regardless of score on risk of bias or study quality assessment. Where a study reported model accuracy but a 95% confidence interval (95% CI) was missing, the confidence interval was conservatively calculated using the formula outlined in the data supplement(S4 Text). If this was not possible the study was excluded from the meta-analysis.

Pooled accuracies (c-statistic’s), with 95% CIs, were calculated for calculating the weighted summary c -statistic under the random effects model (REM). The random-effects meta-analysis model assumes the observed estimates of accuracy vary across studies because of real differences in the accuracy in each study as well as sampling variability (chance). Given differences in case mix and baseline risk, it is expected that the accuracies will be similar but not identical across studies, therefore a REM is a more appropriate than say a fixed effect model. Statistical heterogeneity was assessed using the I2 statistic. Analysis was undertaken in R (R studio) version 3·1·3. The number of validation studies is favoured over sample size. Although larger sample sizes may lead to more precise estimates, with additional, adequately powered (as per inclusion criteria) studies our pooled estimate becomes less bias.

## Results

**Fig 1:**
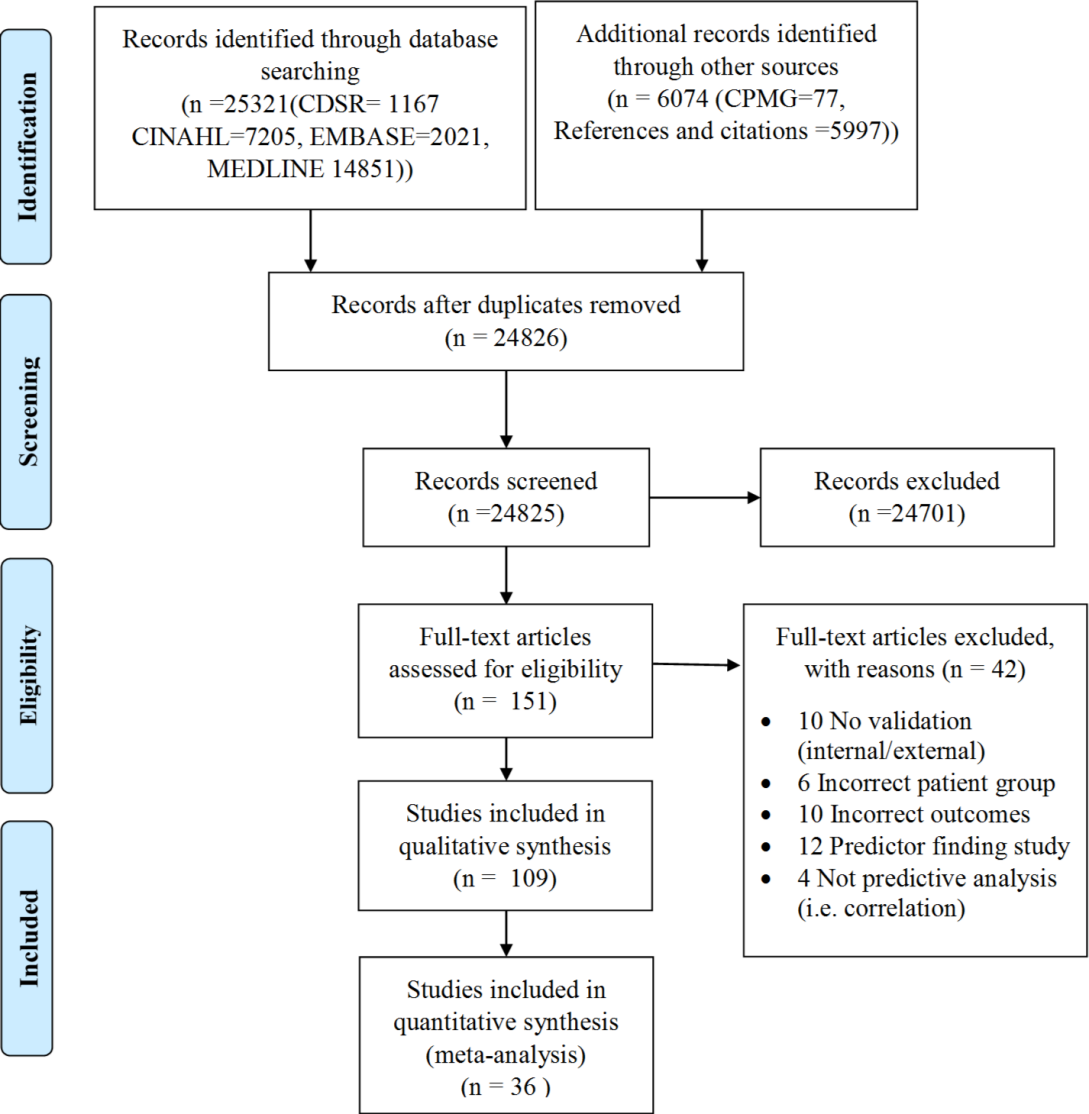
PRISMA diagram.

109 articles meeting inclusion criteria were identified (Fig 1), describing 66 pms estimating stroke functional outcome (mortality n = 27, function n = 28 mortality/function n = 11)(S2 and S3 Tables). The average time to outcome after stroke was three months [7days to ten years] in development/ internal validation cohorts and three months [7 days to 5 years] in models with external validation (S3 Table). Blinded double extractions and assessment demonstrated excellent agreement (κ = 0·81 for selection, κ = 0·75 for risk of bias, κ = 0·83 for CHARMS).

### Qualitative summary of included models using CHARMS domains

#### Sources of data and participants

Data sources included randomised control trial (n = 36) or registry data (n = 31), with only ten pms developed from cohort studies (number of centers range: 1–4, hospital setting N = 9 or community setting N = 1). pms were most often developed from hospital-based populations (31/37 for pms estimating mortality; 31/36 for pms estimating functional outcome; 8/8 for pms estimating mortality/ functional outcome). All pms, except one were developed in populations from developed countries. Sample sizes in development cohorts varied widely, ranging from 107 37 to 274, 98 38 with a mean age of 70 to 75 years. Key characteristics are typically evenly distributed between development and validation cohorts. The proportion of men in cohorts ranged from 46·52% to 59·2%. Many of the identified pms are for ischemic stroke only. However, 27 pms predict outcome in multiple stroke subtypes.

#### Outcome(s)

Measures of functional outcome definition and measurement were the same for all participants in each study. End points were both single, for example mrs >2 at threemonths[28] and combined, for example mortality or mrs >2 at nine months[29] in included pms. No study stated what outcomes were measured without knowledge of candidate predictor variables (i.e. Blinded). No study stated whether candidate predictor variables were part of the outcome (e.g. In panel or consensus diagnosis) in any of the identified models. Time of assessment/death ranges from hospital discharge to one year after stroke for included pms estimating functional outcome and from in hospital to ten years post stroke or more in pms estimating mortality. Pms estimating mortality/ functional outcomes ranged from three months to nine months.

#### Candidate predictors

**Fig 2:**
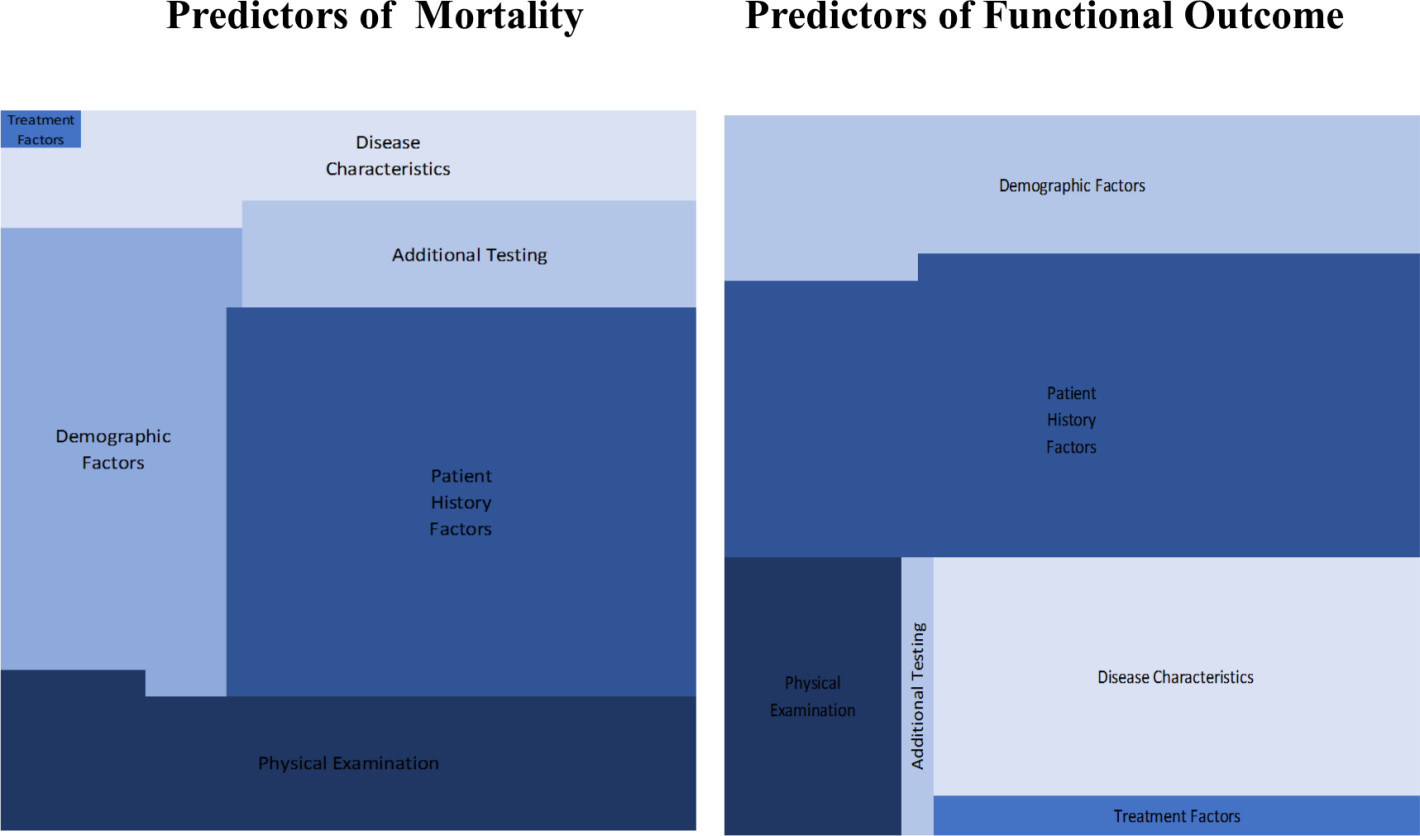
Predictors of mortality and functional outcome.

152 predictor variables of mortality and 195 predictor variables of functional outcome were identified in total. Demographic variables including age, sex, patient history factors such as a history of diabetes, atrial fibrillation or hypertension and dependency prior to stroke were most common (Fig 2). The methods of selection of candidate predictor variables for inclusion in multivariable modelling was not described for many models, where it was described candidate predictor variables were identified by reviewing the literature and expert opinion respectively. Predictor variables during model selection was clearly defined in identified pms, but the measurement method of these predictor variables was often omitted. Timing of predictor measurement (e.g. At patient presentation, at diagnosis or at treatment initiation) was at patient presentation in all identified pms. It was not stated whether researchers assessing predictor variables were blinded to outcome, and to other predictors (where relevant) in any of the identified pms. Although measuring predictor variables on a continuous scale is more accurate, 17 studies used categorical scales where a continuous scale would have been possible.

#### Sample size and missing data

As per inclusion criteria, the sample size was generally considered adequate for identified models. However, none of the included studies considered a sample size calculation. Nine studies were excluded from this review due to inadequate sample size. 50/109 studies report the total number of participants with any missing values (include predictor variables and outcome), but do not specify the number of participants with missing data for each predictor variable or outcome. No model reported investigation regarding quantity or mechanisms of missing data. The handling of missing data is reported in 12 studies, but evidence justifying their decisions is unclear.

#### Model development

Logistic regression (62 models), Cox regression (ten models), General Estimating Equations (4 models), Linear models (2 models) and Data mining (2 models) was used to develop identified pms. The modelling method was not specified for one pm. No study reported whether modelling assumptions were satisfied or any investigations undertaken to test assumptions. Backward (N = 25 models) or forward (N = 20 models) selection methods were used for selection of predictor variables during multivariable modelling where specified (21 not specified). Shrinkage (after estimation or during estimation) of predictor weights or regression coefficients was not described in any of the identified models, although this is not a necessity it is a common way to improve predictions from a regression model[30].

#### Model performance and evaluation

55/109 studies reported calibration using calibration plots, but many did not report calibration slope or 95% confidence interval (N = 20/55). 68/109 studies reported calibration using the Hosmer-Lemeshow test. 16/109 studies did not report calibration. Although most models illustrated discrimination using Receiver Operating Characteristic Curves (ROC) (n = 71), essential data such as AUC and 95% CI are missing in many models. Additionally, it was not stated in any of the identified models if cut-points were chosen a priori. However, we did observe an improvement in reporting of model discrimination and calibration over time. Where model performance was tested in the same setting, data were either randomly split (n = 30) or resampling methods were used (bootstrap n = 39; cross validation n = 1). The discrimination of pms validated in their development population ranged from 0·67[0·60, 0·72] to 0·95[0·91, 0·98]. 64 models were externally validated (temporal n = 22, geographical n = 40, different setting n = 2, different investigators n = 0). None of the authors report model recalibration to these external populations, although the practice is recommended. The discrimination of externally validated models ranged from 0·60[0·57, 0·64] to 0·94[0·91, 0·96]. Several of the identified pms have been updated to simplify, adjust for alternate populations or improve accuracy. [31–35]

### Quantitative summary of included models using meta-analysis

**Fig 3:**
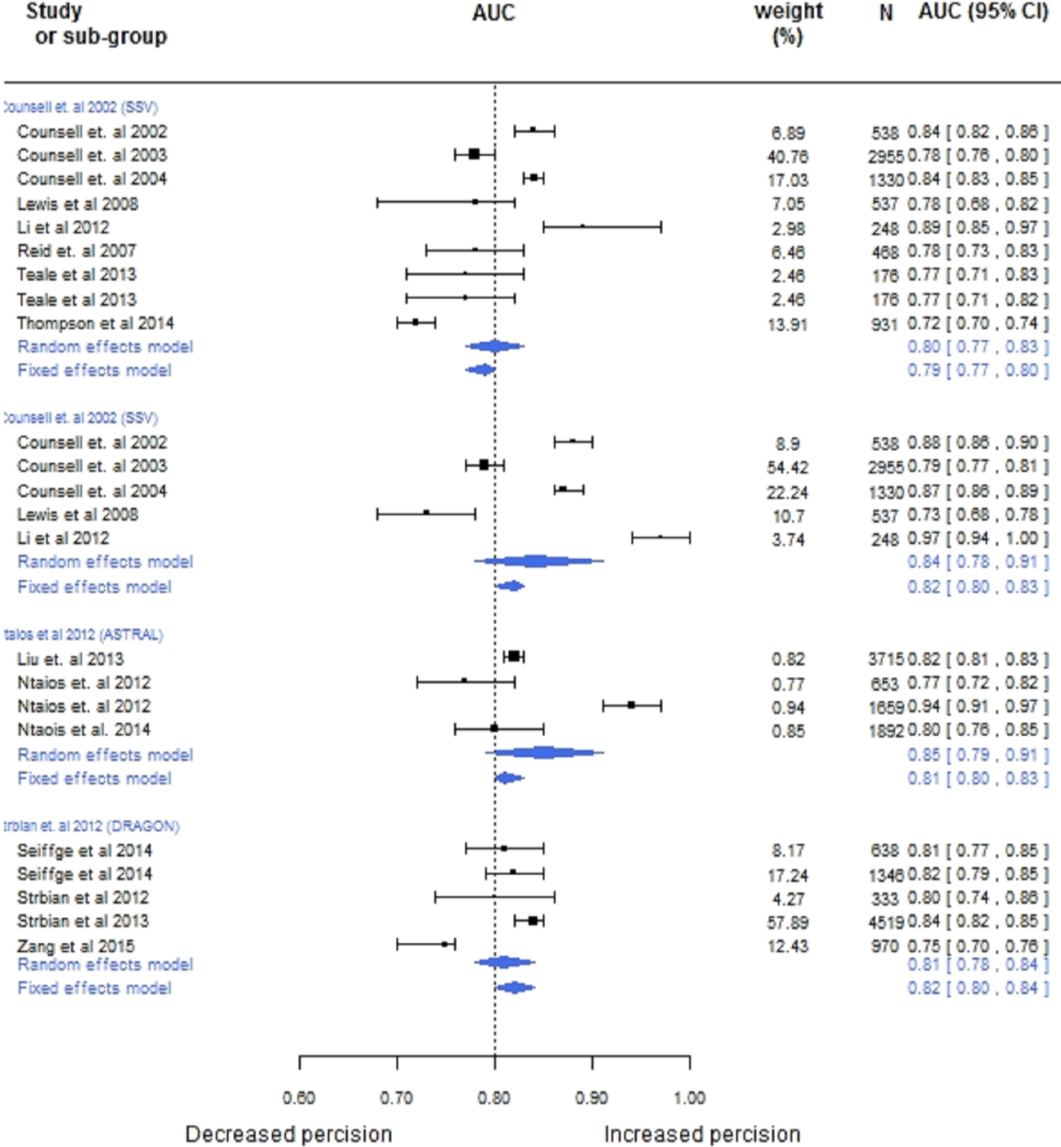
Forest plot AUC (95%CI) values for CPM with ≥4 validation studies.

Internally and externally validated models and models providing c-statistic’s and 95%CI’s or sufficient information to estimate these values (c-statistic/ROC Curve and sample size) were included in the random effects meta-analyses (Internal validation N = 31 models, External validation N = 38). Models with ≥ 4 external validation studies were also meta anlysed and are presented in Fig 3. C-statistics or 95% CI’s were estimated from other information in 51 of the 69 studies included in the meta-analysis.

Pooled accuracy was high for models predicting mrs ≤ 2 at 3 months (0·81[0·80–0·82]) in internal cohorts. In external cohorts models predicting mrs ≤ 2 at 3 months appear to decrease slightly, but confidence intervals overlap (0·79[0·77–0·80]). Models predicting mrs ≤ 1 at 3 months show good accuracy in external cohorts (0·76[0·75–0·78]). Conversely,in models estimating mrs ≥ 5 at 3 months discrimination appears higer in external cohorts compared with internal cohorts (0·79[0·77–0·80] vs. 0·81[0·79–0·83). Models predicting mortality within 30 days of stroke also appear to performed better in external populations (0·83[0·81–0·85] vs. 0·80[0·75–0·85]). However, in both cases this is non conclusive as confidence intervals overlap.The SSV[36] model had the largest number of validation cohorts (nine) and showed good performance in estimating functional outcome (0·80[0·77, 0·83]) and mortality (0·84 [0·78,0·91]) in the random effects model. The ASTRAL[28] and DRAGON[31] Scores also showed high discrimination (0·85[0·79,0·91] and 0·76[0·71,0·82] respectively). Although widely cited, a lack of consistency among outcomes in validation studies identified in our search inhibited meta-analysis of the iscore[32]. There was considerable heterogeneity in validation studies (I2 = 84·32 for ASTRAL[28] I2 = 75·15 for DRAGON[31] score), likely caused by differences in case mix variation or baseline risk. Although, the power of the tests is too low to distinguish chance from real asymmetry (<10 studies) the funnel plot of internally valid models predicting in hospital mortality show a high risk of bias(S1 Fig).

### Appraisal of included models

**Fig 4:**
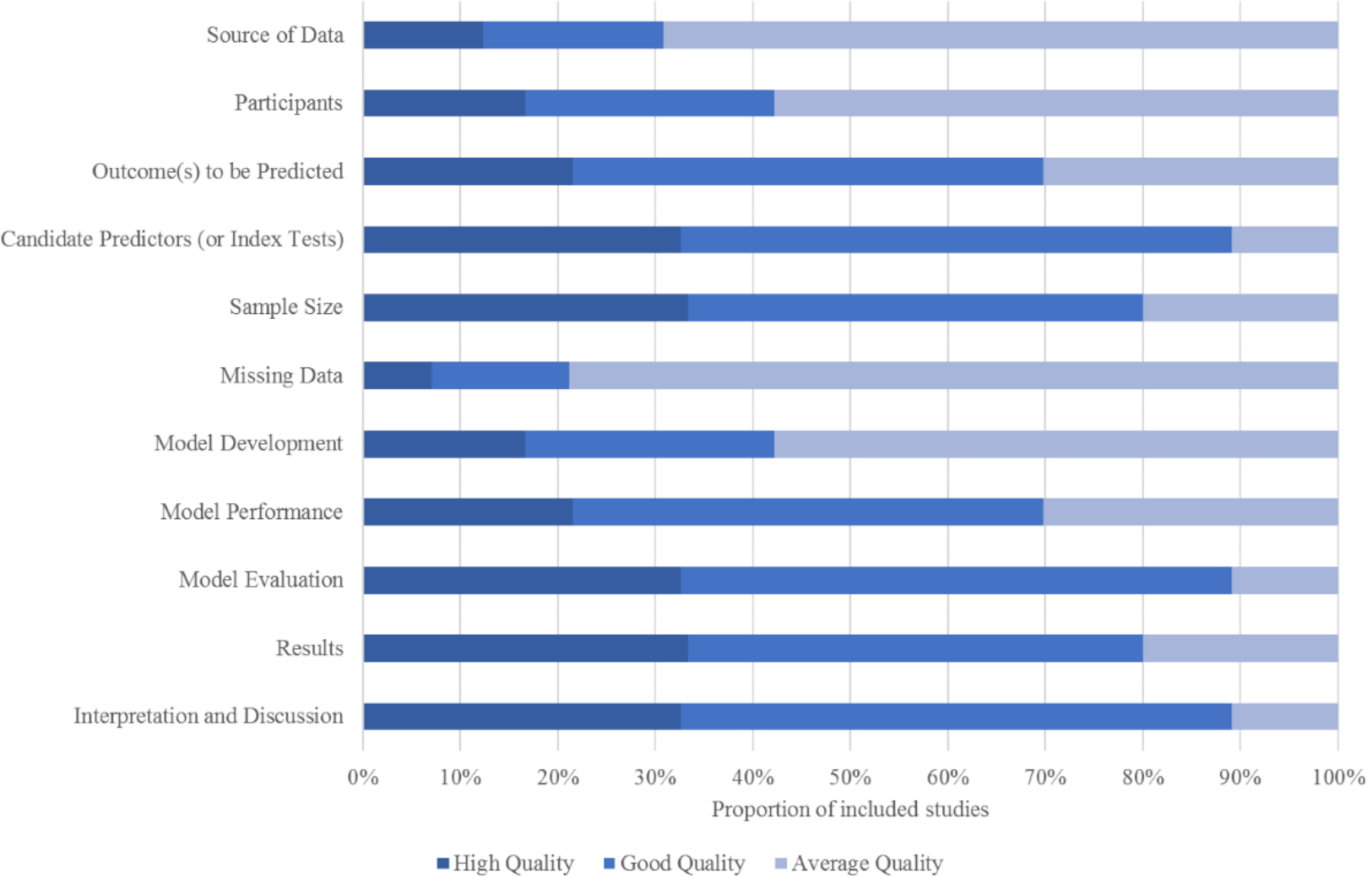
Methodological quality (% across all studies by domain).

**Fig 5:**
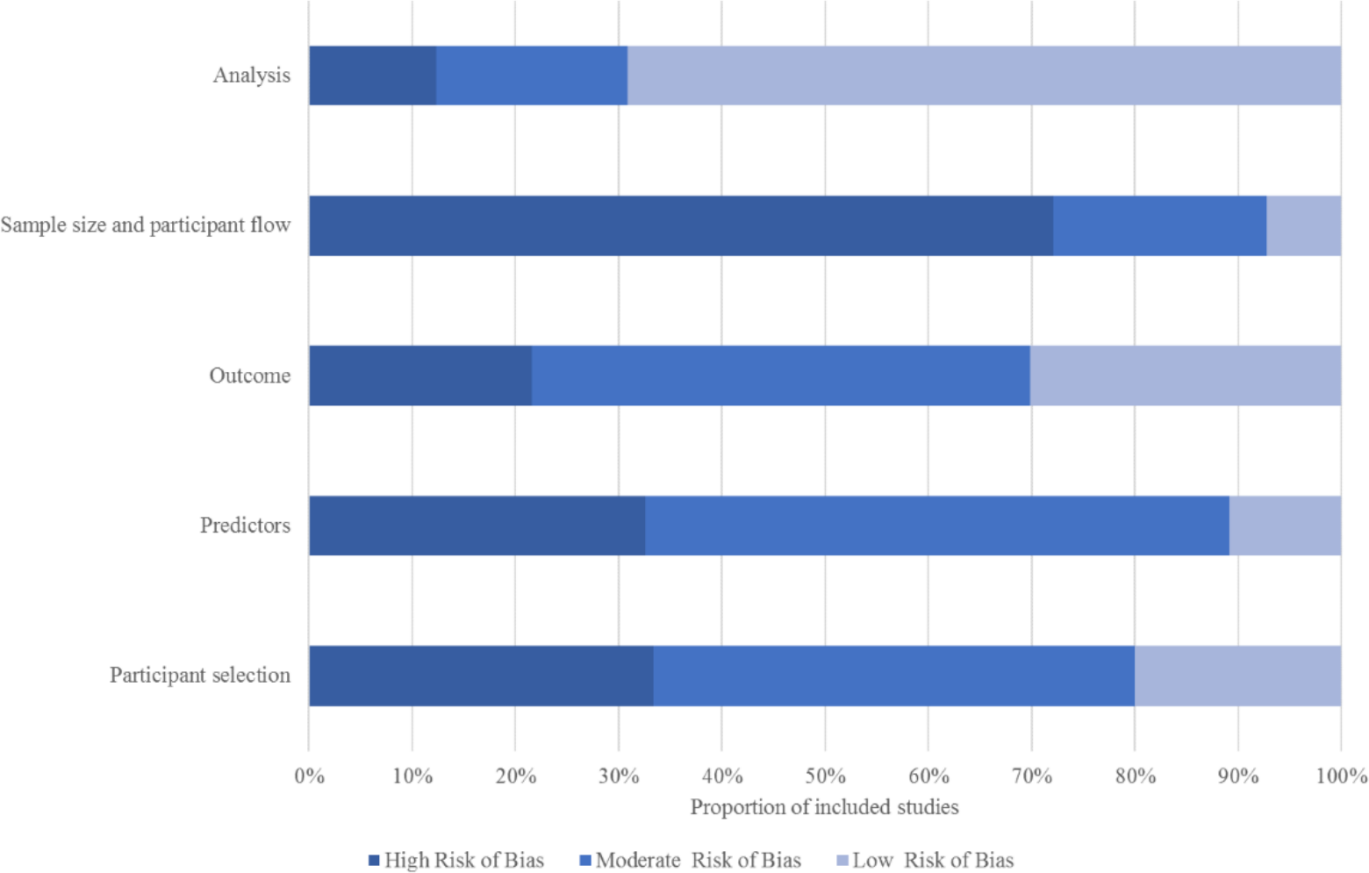
Risk of bias (% across all studies by domain).

As illustrated in Figs 4 and 5 none of the identified pms scored high methodological quality and low risk of bias. However it is important to note, at this point, that all of the included studies pre date the tools used in assessing risk of bias and methodological quality. That uncertainty among researchers in repoirting requirements for prediction research is widely recognized and much of the recent prediction research has focused on improving reporting.

## Discussion

This systematic review has identified 109 studies describing 66 different pms for mortality and/or functional outcome following ischemic stroke. This study assessed pms for methodological quality, generalisability and risk of bias using comprehensive, recent guidelines. Detailed Meta-analyses were performed to estimate pooled accuracies for specific outcomes and where possible for individual predictive models.

In this study, we have observed a clear improvement in methodlogy over time, particularly of model performance. This study also demonstrates that improvements could be made in transparent identification of candidate predictor variables, handling of missing data, controlling for treatment effects, the presentation of results and presentation of the final model. The discrimination of pms varied significantly across outcomes and time points and was typically reduced in external validation cohorts. Interestingly in the thirty-five years of literature, presented in this review, routinely collected factors such as age, sex, disease characteristics (severity, subtype) and comorbidities (diabetes, atrial fibrillation) are consistently identified as the most suitable predictor variables of functional outcome and mortality. We did not identify any impact assessments studies[37], for example, randomised/ non-randomised follow-up studies or cross-sectional studies, or any within study assessments of utility, such as decision curve analysis[38]. However, we are aware of studies comparing the predictive accuracy of the ASTRAL[39], DRAGON[39], Iscore[32]and SEDAN[39] scores with clinical predictions, which provides some evidence that PM could positively impact patient outcome.

Some of the models criticised in previous reviews for lacking assessment of generalisability to external populations are now externally validated and report acceptable accuracies (>0.70). Researchers have increased potential application of their models by examining model validity at different time points after stroke, in different settings (i.e. Hospital vs. Community) and in different patient subgroups. However, for more than half of the models included in this review, there remains a need for studies that focus on further validation using different external cohorts, improving reported accuracies by refining models, and importantly assessing the impact of models in a clinical settings.

Many of the models presented here have the potential to be useful in clinical practice and research. Those wishing to predict stroke outcome are advised to build on previous work, to update and adapt validated models to their specific contexts opposed to designing new ones. For example, for those interested to predict functional outcome: As the SSV[36] model has the most external validations (N = 9) and demonstrates high accuracy in meta-analysis, it is advisable that clinicians/reserchers update this model for their specific context, externally validate the model and assess the impact of using the model on patient outcome opposed to desiging a new model. Similarly, in predicting mortality it may be advisable to adapt the iscore[32] -It has the highest number of validations among models for mortality and boasts good perfomrnace in terms of methodological quality and risk of bias assessments. We stress that external model validation studies alone do not indicate the extent to which the use of such models effect medical decision making or stroke patient outcome and that the effect on decision making and patient outcomes must be evaluated in comparative studies before models are implemented.

Double blind identification and appraisal has ensured this review is reproducible, transparent and reliable. For the first time, the network of the publications for each model are consolidated and evaluated collectively. To our knowledge this is the first review of pms for tertiary prevention in stroke that has accounted for the newly recommended guidelines in prediction and is a comprehensive update of the previous systematic reviews and meta-analyses[40,41]. Arguably, this review is limited by the decision not to contact authors for unreported data. Another limitation is that the criteria used for selecting studies were defined by the same investigators who executed the search and critical appraisal. Future reviewers may also prefer to Meta-analyse using raw patient data to overcome the limitations of study level meta-analysis.

As an assistive tool these PMS may help clinicians make risk based decisions regarding discharge planning and tertiary prevention. When prognosis is ambiguous and the clinician must consider many complex, interacting variables these models provide a ‘second opinion’ as to risk of mortality and likelihood of dependence.

## Summary/Conclusions

In conclusion, this study has identified accurate predictive risk models of mortality and recovery, their usefulness remains unclear. Further external validations and model impact studies to confirm their utility in supporting decision-making are needed. Existing models have much potential. Those wishing to predict stroke outcome are advised to build on previous work, to update and adapt existing models to specifc contexts opposed to designing new ones.

## Supporting information

S1 Text Clinical prediction models for mortality and functional outcome following ischemic stroke: A systematic review and meta-analysis protocol. (PDF)

S2 Text Details of literature search (for existing systematic reviews and meta-analysis of CPM). (DOCX)

S3 Text Details of tailored search strategy. (DOCX)

S4 Text Calculation of confidence intervals for meta-analysis. (DOCX)

S1 Table Charms key items to guide the framing of the review aim, search strategy, and study inclusion and exclusion criteria. (DOCX)

S2 Table Characteristics of included model derivation and/or internal validation studies. (DOCX)

S3 Table Characteristics of included external validation studies. (DOCX)

S4 Table PRISMA 2009 checklist. (DOCX)

S5 Table CHARMS checklist. (DOCX)

S1 Fig Meta analyses of models with internal validation only for mortality in hospital/at discharge. (DOCX)
