# Supplementary material for: Clinical prediction models for mortality and functional outcome following ischemic stroke: A systematic review and meta-analysis
Source: PLoS One. 2018 Jan 29;13(1):e0185402. doi: 10.1371/journal.pone.0185402 (PMC5788336; doi:10.1371/journal.pone.0185402)
Supplement: S1 Text — (PDF) [file pone.0185402.s001.pdf]

King’s Research Portal

Link to publication record in King's Research Portal

*Citation for published version (APA):*
Fahey, M. P. Clinical Prediction Models for mortality and functional outcome following Ischemic Stroke: A Systematic Review and Meta-Analysis Protocol.

**Citing this paper**
Please note that where the full-text provided on King's Research Portal is the Author Accepted Manuscript or Post-Print version this may differ from the final Published version. If citing, it is advised that you check and use the publisher's definitive version for pagination, volume/issue, and date of publication details. And where the final published version is provided on the Research Portal, if citing you are again advised to check the publisher's website for any subsequent corrections.

**General rights**
Copyright and moral rights for the publications made accessible in the Research Portal are retained by the authors and/or other copyright owners and it is a condition of accessing publications that users recognize and abide by the legal requirements associated with these rights.

•Users may download and print one copy of any publication from the Research Portal for the purpose of private study or research.
•You may not further distribute the material or use it for any profit-making activity or commercial gain
•You may freely distribute the URL identifying the publication in the Research Portal

**Take down policy**
If you believe that this document breaches copyright please contact librarypure@kcl.ac.uk providing details, and we will remove access to the work immediately and investigate your claim.

Download date: 29. Jun. 2017

## Any amendments to protocol:

- A prediction model development/validation study specific risk of bias assessment tool was developed in 2016 (Prediction Study Risk of Bias Tool (PROBAST)). Risk of bias assessment was repeated using this tool.

**PROSPERO Format Protocol**

# Clinical Prediction Models for mortality and functional outcome following Ischemic Stroke: A Systematic Review and Meta-Analysis.

## Review Title and Timescale

1. ***Review Title:***

Clinical Prediction Models for mortality and functional outcome following Ischemic Stroke: A Systematic Review and Meta-Analysis

2. ***Original Language Title***

   Not applicable.

3. ***Anticipated or Actual Start Date***

   January 2015

4. ***Anticipated Completion Date***

   March 2015

5. ***Stage of Review at Time of This Submission***

   *Checklist

      The review has not yet started [ ]

## Review Team Details

6. ***Name of contact***

   Ms. Marion Fahey.

**PROSPERO Format Protocol**

*7. Named Contact E-mail*

Marion.fahey@kcl.ac.uk

*8. Named Contact Address*

King's College London

Department of Primary Care and Public Health Sciences

Faculty of Life Sciences & Medicine

9th Floor, Capital House

42 Weston St

LONDON SE1 3QD

*9. Named Contact phone Number*

02078488732

*10. Organisational Affiliation of the Review*

King's College London

http://www.kcl.ac.uk/medicine/index.aspx

*11. Review Team Members and their Organisational Affiliations*

Ms. Elise Crayton, King’s College London, Department of Primary Care and Public Health Sciences

Dr. Abdel Douiri, King’s College London, Department of Primary Care and Public Health Sciences

Professor Charles Wolfe, King’s College London, Department of Primary Care and Public Health Sciences

*12. Funding Sources/Sponsors*

Department of Health and Social Care Research, King’s College London

*13. Conflicts of Interest*

None Known.

***14. Collaborators***

Not applicable.

**Review Methods**

***15. Review question(s)***

What models have been developed to predict mortality after stroke in the mid and long term?

What models have been developed to predict poor functional outcome after stroke in the mid and long term?

What is the quality of the evidence supporting established stroke risk prediction models of death and poor functional outcome in the mid and long term following stroke?

**16. *Searches*:**

*Literature search:*

A multi-method evidence synthesis weighted toward citation and reference list searching utilising validated search filter.

*Meta-analysis:*

Bayesian meta-analysis will be conducted using the Markov Chain Monte Carlo (MCMC) simulation with random effects model.

***17. URL to Search Strategy***

***18. Condition or domain being studied***

The focus of this review is stroke prognostication. 'In clinical medicine, the term prognosis refers to the risk of future health outcomes in people with a given disease or health condition. Prognosis research is thus the investigation of the relations between future outcomes

(endpoints) among people with a given baseline health state (start point) in order to improve health' (Hemingway 2013). The start point in this review is stroke and the endpoint stroke recovery measured proximally as death or disability. The focus of this review is not the resolution of stroke, but rather the prediction of the outcomes of that stroke. This review fits within the broader scope of prognosis research.

Five distinct types of multivariable prediction research have been identified (Bouwmeester 2012): Predictor finding studies, Model development studies without external validation, model development studies with external validation, external validation studies without or with model updating and model impact studies.

A prognostic model is a formal combination of multiple predictors (Identified as any measure that, among people who have had a stroke, is associated with a subsequent increased or decreased risk of death, disability or institutionalisation (Riley, Hayden et al. 2013) from which risks of a specific endpoint can be calculated for individual patients. Other names for a prognostic model include prognostic (or prediction) index or rule, risk (or clinical) prediction model, and predictive model. For an individual with a given state of health (post stroke), a prognostic model converts the combination of predictor values to an estimate of the risk of experiencing a specific endpoint (death, disability or institutionalisation) within a specific period. Ideally this produces an estimate of the absolute risk (absolute probability) of experiencing the endpoint, but it may instead provide a relative risk or risk score (Harrell 2001; Steyerberg 2009; Moons, 2009). As we desire as comprehensive a review as possible for the prognostic accuracy of these models, we will consider all multivariable models, including all relevant populations and outcomes that have been examined. We will thus be using a broad approach to the inclusion criteria.

***19. Participants/ population***

**PROSPERO Format Protocol**

Target patients are individuals who have had an ischemic stroke. Populations for this review will be broadly inclusive, involving any country, both sexes and patients managed in the community or in hospital. Paediatric stroke, secondary stroke or any extremes which do not reflect the general population will be excluded.

***20. Intervention(s), exposure(s)***

This review is intended to identify multivariable formal clinical prediction models estimating risk of poor functional outcome and mortality in the mid and long term following haemorrhagic stroke. Quality assessment criteria will be applied to review the methodical quality of included models.

***21. Comparator(s)/ control***

The various models identified will be compared to recommended best practice. Reviews and Meta-analysis of prognostic studies are relatively new within the field of review and so methods have not been sufficiently standardised or adopted. New research publications are published frequently describing advances in proposed methods (Moons 2014). Thus this review will be informed by current, appropriate guidance (Bouwmeester 2012; Collins 2011; Mallett 2010a; Mallett 2010b; Van Dieren 2012; Steyerberg 2013).

22. ***Types of study to be included initially***

- Prediction model development with internal validation only
- Prediction model development with external validation by independent data
- External model validation with or without model updating
- Impact assessment studies-randomised or non-randomised

This review will focus on multivariable, formal clinical prediction models, excluding studies that investigated a single predictor, test or marker (such as single diagnostic test accuracy or

single prognostic marker studies), studies that investigated only causality between one or more variables and an outcome and predictor finding studies.

***23. Context***

No health care systems, country of origin or place of residence will be excluded a priori. Models that have been developed for use in both the primary and acute care setting will be identified and evaluated.

***24. Primary Outcome(s)***

Mortality risk

Risk of poor functional outcome

***25. Secondary Outcome(s)***

There are no secondary outcomes that will be considered in this review.

***26. Data Extraction (Selection and Coding)***

Selection of studies

Studies will be selected independently and in duplicate by an independent review author (E.C). Disagreement will be resolved by discussion or by a third review author as arbiter(A.D). We will initially screen studies by title and will receive full reports for potentially relevant studies. For these studies, we will use a predefined electronic spreadsheet in conjunction with the Covidence software to assess and document studies for inclusion and exclusion according to the above selection criteria. We will document study selection in a detailed flowchart.

Data extraction and management

Data extraction will be comprehensive and will broadly include the following:

- Data sources (e.g. prospective vs retrospective cohort, nested case-control, case-cohort, sample size).
- Source of participants (e.g. country, facility type, health care system).
- Outcome(s) definitions.
- Candidate predictors (e.g. demographics, stroke severity, pre stroke function).
- Model development (e.g. univariable screening, criteria for prediction selection, statistical software).
- Model performance (e.g. discrimination, calibration).
- Model evaluation (e.g. development and test data sets, external data sets).
- Model interpretation.
- Model impact assessment (Formal assessment or as per model author, generally improved outcomes following stroke, lower incidence of recurrence etc.).

Data extraction will be performed independently and in duplicate by an independent review author (E.C). Disagreement will be resolved by discussion or by involving a third reviewer as arbiter. Should RevMan be adopted to include a template for prognosis studies throughout the duration of this review.

***27. Risk of Bias (Quality) Assessment***

*see ammendments

The QUIPS (QUality In Prognosis Studies) tool has been used successfully by more than 40 prognosis review teams (Hayden 2006; Hayden 2013). Six domains are critical for assessing biases sufficiently large to distort the findings of prognosis research: (1) study participation; (2) study attrition; (3) prognostic factor measurement; (4) outcome

measurement; (5) study confounding; and (6) statistical analysis and reporting. For each domain, three to seven “prompting items” are used to rate the adequacy of reporting by a study as yes, partial, no or unsure; an overall rating for each domain is assigned as high, moderate or low risk of bias. Two review authors will independently complete the QUIPS assessment for each study. We will be guided by previous reports by Bouwmeester et al (Bouwmeester 2012), Collins et al (Collins 2011), Mallet et al (Mallett 2010a; Mallett 2010b), van Dieren et al (van Dieren 2012) and Steyerberg (Steyerberg 2009) . Differences will be resolved by consensus or by referral to a third review author.

GRADE and SOF

The Grades of Recommendation, Assessment, Development and Evaluation (GRADE) framework for judging the quality of evidence has been extended to prognosis factor research. Evidence on prognostic models will be evaluated by six factors that may decrease quality: (1) phase of investigation; (2) study limitations; (3) inconsistency; (4) indirectness; (5) imprecision; and (6) publication bias; and by two factors that may increase quality: (1) moderate or large effect size; and (2) exposure response gradient (Huguet 2013). If a template for ’Summary of findings’ tables is available, prognostic models with GRADE judgement will be displayed. If such a template is not available, a text description of GRADE judgement will be provided.

### *28. Strategy for Data Synthesis*

This broad review investigates evidence on many prognostic models rather than focusing on the predictive accuracy of a single prognostic model. We expect to identify sufficient prognostic models to allow both a qualitative and quantitative overview. This would include assessment of methods, risk of bias, prediction performance (discrimination, calibration) and so forth. We will follow a schema used by van Dieren et al (van Dieren

2012). Tabular displays will be used to show the following for each model: (1) participant population; (2) number of events/sample size; (3) statistical model type; (4) outcome type; (5) number of predictive factors; (6) discrimination; (7) calibration; (8) internal validation method; and (9) presentation format of the model. For prognostic models that have been externally validated, an additional tabular display will be used to show (1) original model name; (2) validation study identifier; (3) external validation participant population; (4) number of events/sample size; (5) discrimination; (6) calibration; and (7) recalibration.

Meta-analysis packages in the R statistical language will be used for meta-analysis and meta-regression.

***29. Analysis of Subgroups or Subsets***

No subgroups or subset analysis will be stated a priori.

## General Information

***30. Type of Review***

Prognostic

***31. Language***

English

***32. Country***

England

***33. Other Registration Details***

Not applicable

***34. Reference and/or URL for Published Protocol***

***35. Dissemination plans***

Results to be published in relevant journals and used for conference presentations.

***36. Keywords***

Systematic Review; Meta-analysis; Stroke; Clinical Prediction Model; Stroke Recovery;

***37. Details of Any Existing Review of the same Topic by the Same Authors***

Not applicable.

***38. Current Review Status***

Ongoing

***39. Any Additional Information***

This review is being undertaken to inform the design of a predictive tool suitable for long term care settings, both of which will contribute to the authors PhD Thesis.

***40. Details of Final Report/Publication(s)***

## References

Bouwmeester W, Zuithoff NP, Mallett S, Geerlings MI, Vergouwe Y, Steyerberg EW, et al. Reporting and methods in clinical prediction research: a systematic review. PLOS Medicine 2012;9(5):1-12.

Collins GS, Mallett S, Omar O, Yu LM. Developing risk prediction models for type 2 diabetes: a systematic review of methodology and reporting. BMC Medicine 2011;9:103.

Debray TP, Koffijberg H, Vergouwe Y, Moons KG, Steyerberg EW. Aggregating published prediction models with individual participant data: a comparison of different approaches. Statistics in Medicine 2012 Oct 15;31(23):2697-712

Debray TP, Moons KG, Ahmed I, Koffijberg H, Riley RD. A framework for developing, implementing, and evaluating clinical prediction models in an individual participant data meta-analysis. Statistics in Medicine 2013;32(18):3158-80

Ettema RG, Peelen LM, Schuurmans MJ, Nierich AP, Kalkman CJ, Moons KG. Prediction models for prolonged intensive care unit stay after cardiac surgery: systematic review and validation study. Circulation 2010 Aug 17;122(7):682-9, 7 p following p 9

Geersing G-J, Bouwmeester W, Zuithoff P, Spijker R, Leeflang M, Moons K. Search filters for finding prognostic and diagnostic prediction studies in MEDLINE to enhance systematic reviews.. PLOS ONE 2012;7(2):e32844.

Hayden JA, Cote P, Bombardier C. Evaluation of the quality of prognosis studies in systematic reviews. Annals of Internal Medicine 2006 Mar 21;144(6):427-37.

Hayden JA, van der Windt DA, Cartwright JL, Cote P, Bombardier C. Assessing bias in studies of prognostic factors. Annals of Internal Medicine 2013 Feb 19;158(4):280

Harrell FE Jr (2001) Regression modeling strategies with applications to linear models, logistic regression, and survival analysis. Springer

Hosmer D, Lemeshow S. Confidence interval estimates of an index of quality performance based on logistic regression. Statistics in Medicine 1995;14(19):2161-72

Huguet A, Hayden JA, Stinson J, McGrath PJ, Chambers CT, Tougas ME, et al. Judging the quality of evidence in reviews of prognostic factor research: adapting the GRADE framework. Systematic Reviews 2013 Sep 5;2(1):71.

Mallett S, Royston P, Dutton S, Waters R, Altman DG. Reporting methods in studies developing prognostic models in cancer: a review. BMC Medicine 2010;8:20.

Mallett S, Royston P, Waters R, Dutton S, Altman DG. Reporting performance of prognostic models in cancer: a review. BMC Medicine 2010;8:21. (b)

Moons KGM, de Groot JAH, Bouwmeester W, Vergouwe Y, Mallett S, Altman DG, et al. Design, critical appraisal and data extraction in systematic reviews of prediction models: the CHARMS checklist. PLoS Medicine 2014;In Press.

Moons KG, Royston P, Vergouwe Y, Grobbee DE, Altman DG (2009) Prognosis and prognostic research: what, why, and how? BMJ 338: b375.

Riley RD, Hayden JA, Steyerberg EW, Moons KG, Abrams K, Kyzas PA, et al. Progress Group. Prognosis Research Strategy (PROGRESS) 2: prognostic factor research. PLOS Medicine 2013;10(2)

Siontis GC, Tzoulaki I, Siontis KC, Ioannidis JP. Comparisons of established risk prediction models for cardiovascular disease: systematic review. BMJ 2012;344

Siregar S, Groenwold RHH, de Heer F, Bots ML, van der Graaf Y, van Herwerden LA. Performance of the original EuroSCORE. European Journal of Cardio-Thoracic Surgery 2012;41(4):746-54

Steyerberg EW (2009) Clinical prediction models: a practical approach to development, validation, and updating. Springer.

Steyerberg EW, Moons KG, van der Windt DA, Hayden JA, Perel P, Schroter S, et al. Progress Group. Prognosis Research Strategy (PROGRESS) 3: prognostic model research. PLOS Medicine 2013;10(2)

Van Dieren S, Beulens JW, Kengne AP, Peelen LM, Rutten GE, Woodward M, et al. Prediction models for the risk of cardiovascular disease in patients with type 2 diabetes: a systematic review. Heart 2012 Mar;98(5):360-9.

van Weijen, D. (2012). The Language of (Future) Scientific Communication. Research Trends, 31. Accessed 22nd December 2014 http://www.researchtrends.com/issue-31-november-2012/the-language-of-future-scientific-communication/
