# Supplementary material for: Clinical prediction models for mortality and functional outcome following ischemic stroke: A systematic review and meta-analysis
Source: PLoS One. 2018 Jan 29;13(1):e0185402. doi: 10.1371/journal.pone.0185402 (PMC5788336; doi:10.1371/journal.pone.0185402)
Supplement: S2 Text — (DOCX) [file pone.0185402.s002.docx]

S2 Text: Details of literature search (for existing systematic reviews and meta-analysis of CPM)

To inform study design the Cochrane Database of Systematic Reviews and Meta-analysis and MEDLINE (PubMed interface) were electronically searched for existing systematic reviews or meta-analysis of CPM for stroke recovery. Publications by the Cochrane Prognosis Methods research group and reference lists of included reviews were hand searched to identify any additional systematic reviews or meta-analysis that may be of interest. Search details and a flow chart summarising the process and results of the literature review can be found below.No restriction was placed on Language or publication date.

**Search details:**

**Last reviewed: September 2015**

MEDLINE (Pubmed)

1. Stroke [MeSH}
2. (Validat*[Text word] OR Predict*[Title] OR Rule*[Text word]) OR (Predict*[Text word] AND (Outcome*[Text word] OR Risk*[Text word] OR Model*[Text word])) OR ((History[Text word] OR Variable[Text word] OR Criteria[Text word] OR Scor*[Text word] OR Characteristic*[Text word] OR Finding*[Text word] OR Factor*[Text word]) AND (Predict*[Text word] OR Model*[Text word] OR Decision*[Text word] OR Identif*[Text word] OR Prognos*[Text word])) OR (Decision*[Text word] AND (Model*[Text word] OR Clinical*[Text word] OR Logistic Models[Mesh:noexp])) OR (Prognostic AND (History OR Variable*[Text word] OR Criteria OR Scor*[Text word] OR Characteristic*[Text word] OR Finding*[Text word] OR Factor*[Text word] OR Model*[Text word])) OR (“Stratification” OR “ROC Curve”[Mesh] OR “Discrimination” OR “Discriminate” OR “c-statistic” OR “c statistic” OR “Area under the curve” OR “AUC” OR “Calibration” OR “Indices” OR “Algorithm” OR “Multivariable”)
3. 1 AND 2

*Limits: Review (filter); Humans; English*

COCHRANE

#1 "stroke":kw (Word variations have been searched)

#2 MeSH descriptor: [Risk Assessment] explode all trees

#3 MeSH descriptor: [Models, Statistical] explode all trees

#4 (((predict* or multicomponent or multivariable) near model*) or (predict* near (outcome* or risk* or model*)) or ((history or variable* or criteria or scor* or characteristic* or finding* or factor* or value*) near (predict* or model* or decision* or identif* or prognos*)) or (decision* near (model* or clinical* or logistic model*)) or (prognostic near (history or variable* or criteria or scor* or characteristic* or finding* or factor* or model*)) or (observ* near (variation or model*))) (Word variations have been searched)

#5 #2 or #3 or #4 not pain

#6 #1 and #5

*Limits: Review (filter); Humans; English*

COCHRANE PROGNOSIS METHODS GROUP

[*http://prognosismethods.cochrane.org/our-publications*](http://prognosismethods.cochrane.org/our-publications)

**PRISMA 2009 Flow Diagram (for existing systematic reviews and meta-analysis of CPM):**


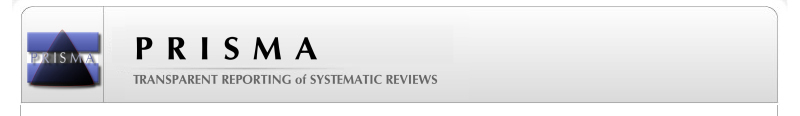


Records identified through database searching
(n =1421) PubMed: n= 1109; CPMG hand search: n= 77

235 All Cochrane Database

Studies included in qualitative synthesis
(n =5)

Full-text articles assessed for eligibility
(n =31)

Records excluded
(n = 1393)

Records screened
(n = 1424)

Records after duplicates removed
(n = 1424)

Additional records identified through other sources
(n =4)

## Identification

## Eligibility

## Included

## Screening

Full-text articles excluded, with reasons (n = 26)

- 4 examined risk of stroke not stroke outcome
- 1 examined models of care not clinical prediction models
- 21 different study design
