# Supplementary material for: Clinical prediction models for mortality and functional outcome following ischemic stroke: A systematic review and meta-analysis
Source: PLoS One. 2018 Jan 29;13(1):e0185402. doi: 10.1371/journal.pone.0185402 (PMC5788336; doi:10.1371/journal.pone.0185402)
Supplement: S3 Text — (DOCX) [file pone.0185402.s003.docx]

S3 Text: Details of tailored search strategy

**Last reviewed: September 2015**

MEDLINE Search Strategy (Ovid interface)

1. (risk assessment/ or statistical model/ or prediction/ or predictive value/ or predictive validity/ or (predict* or validat* or rule* or scor*).ti,ab. or ((predict* or multicomponent or multivariable) adj3 model*).mp. or (predict* adj5 (outcome* or risk* or model*)).ti,ab. or ((history or variable* or criteria or scor* or characteristic* or finding* or factor* or value*) adj5 (predict* or model* or decision* or identif* or prognos*)).ti,ab. or (decision* adj5 (model* or clinical* or logistic model*)).ti,ab. or (prognostic adj5 (history or variable* or criteria or scor* or characteristic* or finding* or factor* or model*)).ti,ab. or (observ* adj3 (variation or model*)).ti,ab.) not pain.mp.
2. stroke/
3. 1 AND 2

*Limits: English language and humans and “all adult(19 plus years)” and medline*

EMBASE Search Strategy (Ovid Interface)

1. (risk assessment/ or statistical model/ or prediction/ or predictive value/ or predictive validity/ or (predict* or validat* or rule* or scor*).ti,ab. or ((predict* or multicomponent or multivariable) adj3 model*).mp. or (predict* adj5 (outcome* or risk* or model*)).ti,ab. or ((history or variable* or criteria or scor* or characteristic* or finding* or factor* or value*) adj5 (predict* or model* or decision* or identif* or prognos*)).ti,ab. or (decision* adj5 (model* or clinical* or logistic model*)).ti,ab. or (prognostic adj5 (history or variable* or criteria or scor* or characteristic* or finding* or factor* or model*)).ti,ab. or (observ* adj3 (variation or model*)).ti,ab.) not pain.mp.

2. stroke.mp. or cerebrovascular accident/

3. 1 and 2

*Limits: Embase and human and english language and (adult <18 to 64 years> or aged <65+ years>))*

CDSR Search Strategy

#1 MeSH descriptor: [Stroke] explode all trees

#2 MeSH descriptor: [Risk Assessment] explode all trees

#3 MeSH descriptor: [Models, Statistical] explode all trees

#4 (((predict* or multicomponent or multivariable) near model*) or (predict* near (outcome* or risk* or model*)) or ((history or variable* or criteria or scor* or characteristic* or finding* or factor* or value*) near (predict* or model* or decision* or identif* or prognos*)) or (decision* near (model* or clinical* or logistic model*)) or (prognostic near (history or variable* or criteria or scor* or characteristic* or finding* or factor* or model*)) or (observ* near (variation or model*))) (Word variations have been searched)

#5 #2 or #3 or #4 not pain

#6 #1 and #5

CINAHL Search Strategy

S1 (MH "Stroke/PR") OR "stroke"

S2 ((MM "Risk Assessment") OR (MM "Models, Statistical") or TI ((predict* or validat* or rule* or scor*) or ((predict* or multicomponent or multivariable) N3 model*) or (predict* N3 (outcome* or risk* or model*)) or ((history or variable* or criteria or scor* or characteristic* or finding* or factor* or value*) N3 (predict* or model* or decision* or identif* or prognos*)) or (decision* N3 (model* or clinical* or logistic model*)) or (prognostic N3 (history or variable* or criteria or scor* or characteristic* or finding* or factor* or model*)) or (observ* N3 (variation or model*))) or AB ((predict* or validat* or rule* or scor*) or ((predict* or multicomponent or multivariable) N3 model*) or (predict* N3 (outcome* or risk* or model*)) or ((history or variable* or criteria or scor* or characteristic* or finding* or factor* or value*) N3 (predict* or model* or decision* or identif* or prognos*)) or (decision* N3 (model* or clinical* or logistic model*)) or (prognostic N3 (history or variable* or criteria or scor* or characteristic* or finding* or factor* or model*)) or (observ* N3 (variation or model*)))) NOT TX pain

*Limits: English Language; Human; Age Groups: All Adult*

WEB OF SCIENCE Search Strategy (excluded due to large number of results)

**search ‘stroke’ returns =72,578 records , ‘ischaemic stroke’ =26,721

CPMG (N=77)

Hand search of all Publications: [*http://prognosismethods.cochrane.org/our-publications*](http://prognosismethods.cochrane.org/our-publications)
