# Supplementary material for: Clinical prediction models for mortality and functional outcome following ischemic stroke: A systematic review and meta-analysis
Source: PLoS One. 2018 Jan 29;13(1):e0185402. doi: 10.1371/journal.pone.0185402 (PMC5788336; doi:10.1371/journal.pone.0185402)
Supplement: S4 Text — (DOCX) [file pone.0185402.s004.docx]

S4 Text: Calculation of confidence intervals for meta-analysis

The following formula was used to estimate unreported confidence intervals in meta-analysis:

$95\%CI=(AUC\pm1.96 SE)$, *where* $SE=\sqrt{\frac{SD}{N}}$ and $SD=\frac{AUC}{2}$

AUC= Area under the curve

SE = Standard Error

N= Sample size

SD = Standard Deviation
