# Supplementary material for: Clinical prediction models for mortality and functional outcome following ischemic stroke: A systematic review and meta-analysis
Source: PLoS One. 2018 Jan 29;13(1):e0185402. doi: 10.1371/journal.pone.0185402 (PMC5788336; doi:10.1371/journal.pone.0185402)
Supplement: S1 Table — (DOCX) [file pone.0185402.s005.docx]

S1 Table: CHARMS key items to guide the framing of the review aim, search strategy, and study inclusion and exclusion criteria

| **Prognostic versus diagnostic prediction model** | Prognostic prediction models |
| --- | --- |
| **Intended scope of the review** | · Models to inform physicians' therapeutic decision making and research |
| **Type of prediction modelling studies** | · Prediction model development studies without external validation  · Prediction model development studies with external validation in independent data  · External model validation studies with or without model updating |
| **Target population to whom the prediction model applies** | Target patients are individuals who have had an ischemic stroke. Populations for this review were broadly inclusive, involving any country, both sexes and patients managed in the community or in hospital. Pediatric stroke, secondary stroke or any extremes which did not reflect the general population were excluded. |
| **Outcome to be predicted** | · Mortality  · Functional outcome (MRS, BI) |
| **Time span of prediction** | Baseline + |
| **Intended moment of using the model** | Model to be used at time of stroke |
