# Supplementary material for: Clinical prediction models for mortality and functional outcome following ischemic stroke: A systematic review and meta-analysis
Source: PLoS One. 2018 Jan 29;13(1):e0185402. doi: 10.1371/journal.pone.0185402 (PMC5788336; doi:10.1371/journal.pone.0185402)
Supplement: S2 Table — (DOCX) [file pone.0185402.s006.docx]

S2 Table: Characteristics of included model derivation and/or internal validation studies

| **Reference** | | **Development population** | **Outcome type** | **n events/n total** | **Statistical model type** | **Number of predictive factors** | **Discrimination** | | | **Calibration (p-value Hosmer-Lemeshow)** | | **Method of Validation** | **Presentation of risk model** | |  |  |  |  |
| --- | --- | --- | --- | --- | --- | --- | --- | --- | --- | --- | --- | --- | --- | --- | --- | --- | --- | --- |
|  |  |  |  |  |  |  | **AUC** | **LCL** | **UCL** |  |  |  | |  | |  |  |  |
| **Ntaios et. al 2012**  **(ASTRAL)** | | Ischaemic stroke patients from ASTRAL  (Jan 2003- July 2010) (Hospital based registry data) | mRs >2 at 3 months | /1645 | Logistic | 6 | 0.85 | 0.82** | 0.88** | 0.43 | | Internal: 2 fold cross validation External: n=4 | | Original model, scoring chart | |  |  |  |
| **O'Donnell et al 2012**  **(Plan score)** | | Ischaemic stroke patients from Registery of the Canadian Stroke Network(RCSN) (Jul 2003- Nov 2008) (Community based registry data(n=11 centres)) | mRs 5- 6 at discharge | 1230/9847  (ND 735/4943; NV 791/4904) | Logistic | 9 | 0.88 | 0.87 | 0.90 | > 0.20 | | Internal: split sample | | Original model, scoring chart | |  |  |  |
|  |  |  | mRs 0-2 at discharge | 1230/9847  (ND xxx/4943; NV xxx/4904) | Logistic | 9 | 0.80 | 0.78 | 0.81 | > 0.20 | |  |  |  |  |  |  |  |
|  |  |  | Mortality at 30 days | 1230/9847  (ND 569/4943; NV 661/4904) | Logistic | 9 | 0.87 | 0.85 | 0.88 | > 0.20 | |  |  |  |  |  |  |  |
|  |  |  | Mortality at 1 year | 1230/9847  (ND 1088/4943; NV 1169/4904) | Logistic | 9 | 0.84 | 0.82 | 0.85 | > 0.20 | |  |  |  |  |  |  |  |
| **O'Donnell et al 2012**  **(Plan-IT score)** | | Ischaemic stroke patients from Registry of the Canadian Stroke Network(RCSN) (Jul 2003- Nov 2008) (Community based registry data(n=11 centres)) | mRs 5- 6 at discharge | 1230/9847  (ND 735/4943; NV 791/4904) | Logistic | 13 | 0.88 | 0.87 | 0.89 | > 0.20 | | Internal: split sample | | Original model | |  |  |  |
|  |  |  | mRs 0-2 at discharge | - | - | - | - | - | - | - | |  |  |  |  |  |  |  |
|  |  |  | Mortality at 30 days | 1230/9847  (ND 569/4943; NV 661/4904) | Logistic | 13 | 0.87 | 0.85 | 0.89 | > 0.20 | |  |  |  |  |  |  |  |
|  |  |  | Mortality at 1 year | 1230/9847  (ND 1088/4943; NV 1169/4904) | Logistic | 13 | 0.84 | 0.83 | 0.86 | > 0.20 | |  |  |  |  |  |  |  |
| **Counsell et. al 2002**  **(Six Simple variables)** | | Acute and subacute stroke of any pathological type patients from Oxfordshire Community Stroke Project(OCSP) (1981-1986) (community based stroke incidence study) | OHS< 3 at 6 months | 134/2398 (ND 51/530; NV 83/1868)£ | Logistic | 6 | - | - | - | | - | External: n=8 | | Original model | |  |  |  |
|  |  |  | Mortality at 30 days | 264/2398 (ND 87/530 ; NV 177/1868)£ | Cox | 6 |  |  |  |  |  |  |  | Original  Original model model | | | | |
| **Seiffge et al 2014** **(Six Simple variables-IVT)** | | Hyper acute stroke patients receiving intravenous thrombolysis (IVT) from observational IVT-databank study, FIN (1995-2010) | mRs 5-6 at 3 months | 180/1346 | Logistic | **5** | 0.807 | 0.774 | 0.838 | **-** | | External: n= | | - | | - | - |  |
| **Kent et al 2006 (TPI)**  **Model 1** | | Ischemic stroke patients treated with rt-PA from 5 RCT databases(NINDS rt-PA Study (Trial 1 and Trial 2),ATLANTIS A, ATLANTIS B, and ECASS 2) (Hospital based) (*date missing)* | mRS ≤1 at 3 month | 773/1983 | Logistic | 13 | 0.79 | 0.77** | 0.82** | Calibration plot | | Internal (bootstrap) | | Original model, web calculator | |  |  |  |
| **Kent et al 2006 (TPI)**  **Model 2** | | Ischemic stroke patients treated with rt-PA from 5 RCT databases(NINDS rt-PA Study (Trial 1 and Trial 2),ATLANTIS A, ATLANTIS B, and ECASS 2) (Hospital based) (*date missing)* | mRS ≥5 at 3 month | 464/1967 | Logistic | 3 | 0.78 | 0.75** | 0.81** | Calibration plot | | Internal (bootstrap) | | Original model, web calculator | |  |  |  |
| **Kent et al 2006**  **(TPI+ ASPECTS)** | | Ischemic stroke patients treated with rt-PA from 5 RCT databases(NINDS rt-PA Study (Trial 1 and Trial 2),ATLANTIS A, ATLANTIS B, and ECASS 2) (Hospital based) (*date missing)* | mRS ≥5 at 3 month | 464/1883 | Logistic | 4 | 0.78 | 0.75** | 0.81** | Calibration plot | | Internal (bootstrap) | | Original model, web calculator | |  |  |  |
| **Kent et al 2015**  **(simplified-TPI)**  **Model 2** | | Ischemic stroke patients treated with rt-PA from 4 RCT databases(NINDS rt-PA Study (Trial 1 and Trial 2),ATLANTIS A and ATLANTIS B) (Hospital based) (*date missing)* | mRS ≤1 at 3 months | 452/1025 | Logistic | 9 (3 item score) | 0.76 | 0.73** | 0.80** | Calibration plot | | Internal (bootstrap) | | Original model | |  |  |  |
| **Kent et al 2015**  **(simplified-TPI)**  **Model 1** | | Ischemic stroke patients treated with rt-PA from 4 RCT databases(NINDS rt-PA Study (Trial 1 and Trial 2),ATLANTIS A and ATLANTIS B) (Hospital based) (*date missing)* | mRS ≤1 at 3 months | 452/1025 | Logistic | 9 (8 item score) | 0.76 | 0.73** | 0.80** | Calibration plot | | Internal (bootstrap) | | Original model | |  |  |  |
| **Kent et al 2015**  **(simplified-TPI)**  **Model 2** | | Ischemic stroke patients treated with rt-PA from 4 RCT databases(NINDS rt-PA Study (Trial 1 and Trial 2),ATLANTIS A and ATLANTIS B) (Hospital based) (*date missing)* | mRS ≤1 at 3 months | 452/1025 | Logistic | 9 (full NIHSS) | 0.78 | 0.73** | 0.80** | Calibration plot | | Internal (bootstrap) | | Original model | |  |  |  |
| **Kent et al 2015**  **(simplified-TPI)**  **Model 3** | | Ischemic stroke patients treated with rt-PA from 4 RCT databases(NINDS rt-PA Study (Trial 1 and Trial 2),ATLANTIS A and ATLANTIS B) (Hospital based) (*date missing)* | mRS ≤2 at 3 months | 595/1205 | Logistic | 9 (3 item score) | 0.78 | 0.73** | 0.80** | Calibration plot | | Internal (bootstrap) | | Original model | |  |  |  |
| **Kent et al 2015**  **(simplified-TPI)**  **Model 4** | | Ischemic stroke patients treated with rt-PA from 4 RCT databases(NINDS rt-PA Study (Trial 1 and Trial 2),ATLANTIS A and ATLANTIS B) (Hospital based) (*date missing)* | mRS ≤2 at 3 months | 595/1205 | Logistic | 9 (8 item score) | 0.77 | 0.74** | 0.81** | Calibration plot | | Internal (bootstrap) | | Original model | |  |  |  |
| **Kent et al 2015**  **(simplified-TPI)**  **Model 5** | | Ischemic stroke patients treated with rt-PA from 4 RCT databases(NINDS rt-PA Study (Trial 1 and Trial 2),ATLANTIS A and ATLANTIS B) (Hospital based) (*date missing)* | mRS ≤2 at 3 months | 595/1205 | Logistic | 9 (full NIHSS) | 0.79 | 0.74** | 0.81** | Calibration plot | | Internal (bootstrap) | | Original model | |  |  |  |
| **Kent et al 2015**  **(simplified-TPI)**  **Model 6** | | Ischemic stroke patients treated with rt-PA from 4 RCT databases(NINDS rt-PA Study (Trial 1 and Trial 2),ATLANTIS A and ATLANTIS B) (Hospital based) (*date missing)* | mRS ≥5 at 3 months | 250/1205 | Logistic | 3 (3 item score) | 0.76 | 0.73** | 0.80** | Calibration plot | | Internal (bootstrap) | | Original model | |  |  |  |
| **Kent et al 2015**  **(simplified-TPI) Model 7** | | Ischemic stroke patients treated with rt-PA from 4 RCT databases(NINDS rt-PA Study (Trial 1 and Trial 2),ATLANTIS A and ATLANTIS B) (Hospital based) (*date missing)* | mRS ≥5 at 3 months | 250/1205 | Logistic | 3 (8 item score) | 0.75 | 0.72** | 0.79** | Calibration plot | | Internal (bootstrap) | | Original model | |  |  |  |
| **Kent et al 2015**  **(simplified-TPI)**  **Model 8** | | Ischemic stroke patients treated with rt-PA from 4 RCT databases(NINDS rt-PA Study (Trial 1 and Trial 2),ATLANTIS A and ATLANTIS B) (Hospital based) (*date missing)* | mRS ≥5 at 3 months | 250/1205 | Logistic | 3 (full NIHSS) | 0.77 | 0.74** | 0.81** | Calibration plot | | Internal (bootstrap) | | Original model | |  |  |  |
| **Strbian et al 2012**  **(DRAGON Score)** | | Ischemic stroke patients treated with IV alteplase at the Helsinki University Central Hospital, Helsinki, Finland (Hospital based) (*missing* 1995 - Sep 2010) | mRS 0 –2 at 3 months | 798/1319 | Logistic | 6 | 0.84 | 0.80 | 0.87 | - | | Internal (bootstrap) | | Original model, scoring chart | |  |  |  |
|  |  |  | mRS 3– 4 at 3 months | 339/1319 | Logistic | 6 | 0.84 | 0.80 | 0.87 | - | | Internal (bootstrap) | | Original model, scoring chart | |  |  |  |
|  |  |  | mRS ≥5at 3 months | 182/1319 | Logistic | 6 | 0.84 | 0.80 | 0.87 | - | | Internal (bootstrap) | | Original model, scoring chart | |  |  |  |
| **Turc et al 2013** **(MRI- DRAGON)** | | Ischemic stroke patients treated with IV alteplase from retrospective single-center cohort, FRA  (Hospital based) (Nov 2003 - Feb 2012) | mRS >2 at 3 months | 98/228 | Logistic | 6 | 0.83 | 0.78 | 0.88 | - | | Internal (bootstrap) | | Original model, scoring chart | |  |  |  |
| **Asadi et al 2014** | | Acute Ischemic stroke patients treated by endovascular intervention from prospective database (Hospital based) (*missing*) | mRS at 3 months | n/a/107 | Machine Learning | - | - | - | - | - | | - | | - | |  |  |  |
| **Weimar et al 2002**  **Model 1** | | Ischemic stroke patients from Database of the German Stroke Foundation , DEU (1998-1999) (Hospital based (n=23)) | Barthel Index ≥95 vs Barthel Index <95 or death (after 100 days) | 1025/1754 vs 729/1754 | Logistic | 11 | 0.79 | 0.76 | 0.82 | Calibration plot | | internal  External (n=1) | | original model | |  |  |  |
| **Weimar et al 2002**  **Model 2** | | Ischemic stroke patients from Database of the German Stroke Foundation , DEU (1998-1999) (Hospital based (n=23)) | Mortality at 100 days | 166/1754 | Logistic | 3 | 0.79* | 0.76 | 0.82 | Calibration plot | | internal | | original model | |  |  |  |
| **Johnston et al 2000**  **Model 1** | | patients with acute ischemic stroke (RANTTAS), USA (Hospital based-27 centres)) (May 1993-Dec 1994) | BI >95 at 3 months | 125/229 | Logistic | 7 | 0.84 (combined) | 0.76 | 0.92 | Calibration plot | | Internal -bootstrap | | original model | |  |  |  |
| **Johnston et al 2000**  **Model 2** | | patients with acute ischemic stroke (RANTTAS), USA (Hospital based-27 centres)) (May 1993-Dec 1994) | GOS =1 at 3 months | 108/229 | Logistic | 7 | 0.84 (combined) | 0.76 | 0.92 | Calibration plot | | Internal -bootstrap | | original model | |  |  |  |
| **Johnston et al 2000**  **Model 3** | | patients with acute ischemic stroke (RANTTAS), USA (Hospital based-27 centres)) (May 1993-Dec 1994) | NIHSS ≤1 at 3 months | 94/221 | Logistic | 7 | 0.87 (combined) | 0.79 | 0.95 | Calibration plot | | Internal -bootstrap | | original model | |  |  |  |
| **Johnston et al 2000**  **Model 4** | | patients with acute ischemic stroke (RANTTAS), USA (Hospital based-27 centres)) (May 1993-Dec 1994) | GOS >2at 3 months | 77/229 | Logistic | 7 | 0.87 (combined) | 0.79 | 0.95 | Calibration plot | | Internal -bootstrap | | original model | |  |  |  |
| **Johnston et al 2000**  **Model 5** | | patients with acute ischemic stroke (RANTTAS), USA (Hospital based-27 centres)) (May 1993-Dec 1994) | BI ≤60 or death at 3 months | 70/229 | Logistic | 7 | 0.88 (combined) | 0.80 | 0.96 | Calibration plot | | Internal -bootstrap | | original model | |  |  |  |
| **Johnston et al 2000**  **Model 6** | | patients with acute ischemic stroke (RANTTAS), USA (Hospital based-27 centres)) (May 1993-Dec 1994) | NIHSS ≥ 20 or death at 3 months | 36/221 | Logistic | 7 | 0.79 (combined) | 0.71 | 0.87 | Calibration plot | | Internal -bootstrap | | original model | |  |  |  |
| **Johnston et al 2007**  **Model 1** | | Ischemic stroke patients from the GAIN Americas andInternational and citicoline 010 and 018 clinical trials  (Hospital based)  (date missing) | BI ≥ 95 at 3 months | 123/382 | Logistic | 5 | 0.794 | 0.731** | 0.857** | Calibration plot | | Internal: bootstrap External: n=1 | | original model | |  |  |  |
| **Johnston et al 2007**  **Model 2** | | Ischemic stroke patients from the GAIN Americas andInternational and citicoline 010 and 018 clinical trials  (Hospital based)  (date missing) | mRs ≤1 at 3 months | 75/382 | Logistic | 5 | 0.776 | 0.714** | 0.838** | Calibration plot | | Internal: bootstrap External: n=1 | | original model | |  |  |  |
| **Johnston et al 2007**  **Model 3** | | Ischemic stroke patients from the GAIN Americas andInternational and citicoline 010 and 018 clinical trials  (Hospital based)  (date missing) | NIHSS ≤1 at 3 months | 74/382 | Logistic | 5 | 0.761 | 0.699** | 0.823** | Calibration plot | | Internal: bootstrap External: n=1 | | original model | |  |  |  |
| **Johnston et al 2007**  **Model 4** | | Ischemic stroke patients from the GAIN Americas andInternational and citicoline 010 and 018 clinical trials  (Hospital based)  (date missing) | mRs ≥5 at 3 months | 87/382 | Logistic | 5 | 0.790 | 0.727** | 0.853** | Calibration plot | | Internal: bootstrap External: n=1 | | original model | |  |  |  |
| **Johnston et al 2007**  **Model 5** | | Ischemic stroke patients from the GAIN Americas andInternational and citicoline 010 and 018 clinical trials  (Hospital based)  (date missing) | BI ≤60 or death at 3 months | 174/382 | Logistic | 5 | 0.792 | 0.729** | 0.855** | Calibration plot | | Internal: bootstrap External: n=1 | | original model | |  |  |  |
| **Johnston et al 2007**  **Model 6** | | Ischemic stroke patients from the GAIN Americas andInternational and citicoline 010 and 018 clinical trials  (Hospital based)  (date missing) | NIHSS ≥ 15 or death at 3 months | 94/382 | Logistic | 5 | 0.825 | 0.761** | 0.889** | Calibration plot | | Internal: bootstrap External: n=1 | | original model | |  |  |  |
| **Johnston et al 2007**  **Model 7** | | Ischemic stroke patients from the GAIN Americas andInternational and citicoline 010 and 018 clinical trials  (Hospital based)  (date missing) | BI ≥ 95 at 3 months | 123/382 | Logistic | 8 | 0.803 | 0.739** | 0.867** | Calibration plot | | Internal: bootstrap External: n=1 | | original model | |  |  |  |
| **Johnston et al 2007**  **Model 8** | | Ischemic stroke patients from the GAIN Americas andInternational and citicoline 010 and 018 clinical trials  (Hospital based)  (date missing) | mRs ≤1 at 3 months | 75/382 | Logistic | 8 | 0.795 | 0.732** | 0.858** | Calibration plot | | Internal: bootstrap External: n=1 | | original model | |  |  |  |
| **Johnston et al 2007**  **Model 9** | | Ischemic stroke patients from the GAIN Americas andInternational and citicoline 010 and 018 clinical trials  (Hospital based)  (date missing) | NIHSS ≤1 at 3 months | 74/382 | Logistic | 8 | 0.802 | 0.738** | 0.866** | Calibration plot | | Internal: bootstrap External: n=1 | | original model | |  |  |  |
| **Johnston et al 2007**  **Model 10** | | Ischemic stroke patients from the GAIN Americas andInternational and citicoline 010 and 018 clinical trials  (Hospital based)  (date missing) | mRs ≥5 at 3 months | 87/382 | Logistic | 8 | 0.811 | 0.747** | 0.875** | Calibration plot | | Internal: bootstrap External: n=1 | | original model | |  |  |  |
| **Johnston et al 2007**  **Model 11** | | Ischemic stroke patients from the GAIN Americas andInternational and citicoline 010 and 018 clinical trials  (Hospital based)  (date missing) | BI ≤60 or death at 3 months | 174/382 | Logistic | 8 | 0.808 | 0.744** | 0.872** | Calibration plot | | Internal: bootstrap External: n=1 | | original model | |  |  |  |
| **Johnston et al 2007**  **Model 12** | | Ischemic stroke patients from the GAIN Americas andInternational and citicoline 010 and 018 clinical trials  (Hospital based)  (date missing) | NIHSS ≥ 15 or death at 3 months | 94/382 | Logistic | 8 | 0.837 | 0.772** | 0.902** | Calibration plot | | Internal: bootstrap External: n=1 | | original model | |  |  |  |
| **Reid et al 2011**  **Model 1** | | Acute ischaemic and haemorrhagic stroke patients (Hospital based) (2001-2002) | mRs≤2 at 6 months | 228/538 *ischemic and haemorrhagic | Logistic | 4 | 0.88 | 0.83 | 0.94 | Calibration plot | | Internal -bootstrap | | original model | |  |  |  |
| **Reid et al 2011**  **Model 2** | | acute ischaemic and haemorrhagic stroke patients (Hospital based) (2001-2002) | mRs≤2 at 6 months | 228/538 *ischemic and haemorrhagic | Logistic | 5 | 0.88 | 0.82 | 0.93 | Calibration plot | | Internal -bootstrap external validation | | original model | |  |  |  |
| **Reid et al 2011**  **Model 3** | | acute ischaemic and haemorrhagic stroke patients (Hospital based) (2001-2002) | mRs≤2 at 6 months | 228/538 *ischemic and haemorrhagic | Logistic | 6 | 0.90 | 0.84 | 0.96 | Calibration plot | | Internal bootstrap | | original model | |  |  |  |
| **Ji et al 2014**  **(DFS-AIS)**  **Model 1** | Acute ischemic stroke patients from CNSR (Hospital based- 132 hospitals) (Sep 2007 - Aug 2008) | | mRs ≤2 at discharge | 8160/12026 (ND*7215; NV*4,811) | Logistic | 7 | 0.80 | 0.77 | 0.83 | Calibration plot | | internal -split sample | | Original model, web calculator | |  |  |  |
| **Ji et al 2014**  **(DFS-AIS)**  **Model 2** | Acute ischemic stroke patients from CNSR (Hospital based- 132 hospitals) (Sep 2007 - Aug 2008) | | mRs ≤2 at 3 months | 8160/12026 (ND*7215;NV*4,811) | Logistic | 9 | 0.81 | 0.80 | 0.82 | Calibration plot | | internal -split sample | | Original model, web calculator | |  |  |  |
| **Ji et al 2014**  **(DFS-AIS)**  **Model 2** | Acute ischemic stroke patients from CNSR (Hospital based- 132 hospitals) (Sep 2007 - Aug 2008) | | mRs ≤2 at 6 months | 8050/12026 (ND*7215; NV*4,811) | Logistic | 9 | 0.81 | 0.81 | 0.82 | Calibration plot | | internal -split sample | | Original model, web calculator | |  |  |  |
| **Ji et al 2014**  **(DFS-AIS)**  **Model 2** | Acute ischemic stroke patients from CNSR (Hospital based- 132 hospitals) (Sep 2007 - Aug 2008) | | mRs ≤2 at 1 year | 8047/12026 (ND*7215; NV*4,811) | Logistic | 9 | 0.82 | 0.81 | 0.82 | Calibration plot | | internal -split sample | | Original model, web calculator | |  |  |  |
| **Flint et al 2010** **(THRIVE)** ***model with 2 predictors does not meet IC*** | | Ischemic stroke patients form MERCI and Multi MERCI RCT, USA (Hospital based) (date *missing)* | mrs 0-2 at 3 months | (*missing)*/305 | Logistic | 3 | 0.709 | 0.64 | 0.78 | - | | External | | Original model, scoring system | |  |  |  |
|  |  |  | Mortality at 3 months | (*missing)*/305 | Logistic | 3 | 0.709 | 0.64 | 0.78 | - | | External | | Original model, scoring system | |  |  |  |
| **Allen 1984**  **(Guy's Score)** | | Acute stroke patients under 76yrs, GB (Sept 1979 - Sept 1981) (Hospital based) | author defined Independence at 2 months | 87/137 | Discriminant function analysis | 4 | - | - | - | - | | External | | Original model, scoring system | |  |  |  |
|  |  |  | author defined Independence at 6 months | 80/128 | Discriminant function analysis | 4 | - | - | - | - | | External | | Original model, scoring system | |  |  |  |
| **LeGall et al 1984** **(SAPS I)** | | ICU Patients, FRA (Hospital based(n=8))  (date *missing)* | mortality at discharge | 185**/679 | *missing* | 14 | external validation only(not developed in stroke population) | | | | | external validation only(not developed in stroke population) | | - | |  |  |  |
| **LeGall et al 1993** **(SAPS II)** | | ICU Patients, 12 countries (Hospital based(n=137)) (Sept 1991 - Feb 1992) | mortality at discharge | 2867/13152 (ND 1836/8369; NV 1031**/4783) | Logistic | 17 | external validation only(not developed in stroke population) | | | | | external validation only(not developed in stroke population) | | scoring sheet | |  |  |  |
| **Birkner et al 2007** | | Stroke patients from MGIMS, IN (Hospital based) (Dec 1999 - Mar 2001) | mortality at 30 days | 124/ 175 (n= 117 IS only) | POLYCLASS logistic regression | 4 | 0.93 | 0.90 | 0.97 | - | | internal (cross validation) | | original model | |  |  |  |
| **Saposnik et. al 2011**  **(IScore)** | | Ischaemic stroke patients from RCSN  (2003-2008)  (Community based) | Mortality at 30 days | 1513/12262 (ND 1004/8223; NV 509/4039) | Logistic | 8 | 0.85 | 0.83** | 0.87** | 0.554 | | internal -split sample | | Original model, scoring chart | |  |  |  |
|  |  |  | Mortality at 1 year | 2777/12262 (ND 1853/8223: NV 924/4039) | Logistic | 8 | 0.84 | 0.86** | 0.82** | 0.554 | | internal -split sample | | Original model, scoring chart | |  |  |  |
| **Anderson et al 1994** | | Acute stroke patients from PCSS, AUS (Feb 1989 - Aug 1990) (Population based) | Mortality at 1 year | 97/349 (IS only) | Cox | 4 | - | - | - | - | | internal -split sample | | Original model | |  |  |  |
| **Ringleb at al 2004**  **(ESRS)** | | Ischemic stroke patients from the CAPRIE trial(RCT) (*missing* 1992- *missing* 1996) (Hospital based) | Combined: Recurrent stroke, myocardial infarction, or cardiovascular death at 3 years | 994**/4496** | Cox | 16 | *external validation only* | | | | | - | | Original model | |  |  |  |
| **Kernan et al 1991** **SPI I** | | Patients with carotid transient ischemia or minor stroke, Cohort study, USA (Jan 1984- Feb 1987) (Hospital based) | Combined: stroke or mortality at 2 years | 17/142(mortality only) | Cox | 5 | - | - | - | - | | External (N=1) | | Original model | |  |  |  |
| **Kernan et al 2000** **SPI II** | | Postmenopausal women with TIA or nondisabling stroke form WEST RCT, USA (Hosital based(n=20)) (1981-1985) | Combined: stroke or mortality at 2 years | 525 | Cox | 7 | *external validation only* | | | | | External (N=1) | | original model | |  |  |  |
| **Myint et al 2014**  **(SOAR)** | | Acute stroke patients 3 stroke registries, GBR (Hospital based)  (Aintree (2005–2010), Newcastle (2000–2005), and Norwich (1997–2010)) | 7 day mortality | 1161** /12355 | Logistic | 4 | 0·79 | 0.78 | 0.80 | Calibration plot | | Internal bootstrap External (n=1) | | Original model 8-point score | |  |  |  |
|  |  |  | In-hospital mortality | 2457**/12355 | Logistic | 4 | 0·79 | 0.78 | 0.81 | Calibration plot | | Internal bootstrap External (n=1) | | Original model 8-point score | |  |  |  |
| **Van Wijk et al 2005**  **(LLACI)**  **Model 1** | | Patients with TIA or minor ischaemic stroke from TIA trial, NLD (Hospital based (n=24)) (Feb 1986- Mar 1989) | Mortality after 10 years or more | Mortality all causes 1489/2362 **email for ischemic only numbers | Cox | 7 | 0·81 | 0·79 | 0·83 | - | | external(n=1) | | original model | |  |  |  |
| **Van Wijk et al 2005**  **(LLACI)**  **Model 2** | | Patients with TIA or minor ischaemic stroke from TIA trial, NLD (Hospital based (n=24)) (Feb 1986- Mar 1989) | Mortality after 10 years or more | Mortality all causes 1489/2362 **email for ischemic only numbers | Cox | 9 | 0·82 | 0·80 | 0·83 | - | | external(n=1) | | original model | |  |  |  |
| **Van Wijk et al 2005**  **(LLACI)**  **Model 3** | | Patients with TIA or minor ischaemic stroke from TIA trial, NLD (Hospital based (n=24)) (Feb 1986- Mar 1989) | Mortality after 10 years or more | Mortality all causes 1489/2362 **email for ischemic only numbers | Cox | 13 | 0·83 | 0·81 | 0·84 | - | | external(n=1) | | original model | |  |  |  |
| **Smith et al 2010** **(GWTG)**  **Model 1** | | Ischemic stroke patients from GWTG database, USA (Hospital based (n=1306)) (Oct 2001-Dec 2007) | in-hospital mortality | 15143/274988 | Logistic | 12 | 0.72 | 0.718** | 0.722** | <0.001 | | Internal (split sample) External (n=1) | | original model | |  |  |  |
| **Smith et al 2010** **(GWTG)**  **Model 2** | | Ischemic stroke patients from GWTG database, USA (Hospital based (n=1306)) (Oct 2001-Dec 2007) | in-hospital mortality | 5667**/109187 | Logistic | 9 | 0.85 | 0.846** | 0.854** | <0.001 | | Internal (split sample) External (n=1) | | original model | |  |  |  |
| **Smith et al 2013** **(GWTG)**  **Model 1** | | Stroke patients from GWTG database, USA (Hospital based (n=1306)) (Oct 2001-Dec 2007) | in-hospital mortality | 28283/ 33865  (ND missing /200319; NV missing /133546) | Logistic | 13 | 0.72 (IS only) | 0.713 | 0.727 | <0.001 | | Internal (split sample) | | prediction tool | |  |  |  |
| **Smith et al 2013** **(GWTG)**  **Model 2** | | Stroke patients from GWTG database, USA (Hospital based (n=1306)) (Oct 2001-Dec 2007) | in-hospital mortality | Missing /123 916  ND misisng/ 74 278; Nv misisng/ 49 483 | Logistic | 14 | 0.84 (IS only) | 0.844 | 0.836 | <0.001 | | Internal (split sample) | | prediction tool | |  |  |  |
| **Knaus et al 1985**  **(APACHE II)** | | Patients from medical, surgical and mixed ICU's, USA (Hospital based (n=13)) (*missing* 1979- *missing* 1982) | In hospital mortality | 993/5030 | Logistic | 12 | external validation only(not developed in stroke population) | | | | | - | | original model | |  |  |  |
| **Zimmerman et al 2006**  **(APACHE IV)** | | ICU patients from observational cohort, USA (Hospital based (n=104) (Jan 2002 - Dec 2003) | In hospital mortality | 72,249**/110558 (ND 66302**/66270;NV 5975**/44288) | Logistic | 142 | external validation only(not developed in stroke population) | | | | | - | | original model | |  |  |  |
| **Bray et al 2014**  **Model 1** | | patients with ischemic stroke or primary ICH in England and Wales (SSNAP) (hospital based(N=162 )) (Jan-Jun 2013) | Mortality at 30 days | 3368/27169 (ND 1188/9000;NV 2180/18169) | GEM | 4 | 0.86 *IS only | 0.85 | 0.97 | Calibration plot | | Internal(split sample) and external(n=1) | | original model | |  |  |  |
| **Bray et al 2014**  **Model 2** | | patients with ischemic stroke or primary ICH in England and Wales (SSNAP) (hospital based(N=162 )) (Jan-Jun 2013) | Mortality at 30 days | 3368/27169 (ND 1188/9000;NV 2180/18169) | GEM | 4 (NIHSS consciousness only) | 0.81*IS only | 0.80 | 0.82 | Calibration plot | | Internal(split sample) and external(n=1) | | original model | |  |  |  |
| **Lee et al 2013** | | Ischemic stroke patients Japanese administrative data, JPN (Hospital based (N=176) (Jul 2010-Jun 2011) | 7 day mortality | 536/21445 (ND 280/10,774;NV 256/10,671) | Logistic | 8 | 0.90 | 0.89** | 0.91** | 0.72 | | internal- split sample | | original model | |  |  |  |
|  |  |  | Mortality at 30 days | 944/21445 (ND 485/10,774 ;NV 470/10,671) | Logistic | 8 | 0.87 | 0.86** | 0.88** | 0.28 | | internal- split sample | | original model | |  |  |  |
|  |  |  | In hospital mortality | 1480/21445 (ND 965/10,774;NV 726/10,671 ) | Logistic | 8 | 0.88 | 0.86** | 0.89** | 0.12 | | internal- split sample | | original model | |  |  |  |
| **Wang 2001** | | patients with acute ischemic stroke Australia (Hospital based) (Jul 1995-Jun 1997) | Mortality at 30 days | 45/440 (ND 22/223; NV 23/217) | Cox | 5 | 0.92 | 0.83 | 1.00 | 57%, 97% and 68% in validation | | internal | | original model | |  |  |  |
| **Wang 2003** | | participants diagnosed with acute ischaemic stroke (Australia) (Hospital based) (Jul 1995-Jun 1997) | Mortality at 1 year | 48/440  (ND 48/223; NV /217) | Cox | 8 | Calculate from graph | - | - | - | | internal -split sample validation External: n=1 | | Original model, scoring system | |  |  |  |
| **Solberg et al 2007**  **Model 1** | | Acute stroke patients >60 years, NOR (Hospital based) (Jan 1993- Nov 1999) | 1 year mortality | 734  (ND;440 NV;294) | logistic | 3 | 0.71 | 0.65 | 0.77 | 0.773 | | Internal (split sample) | | original model | |  |  |  |
| **Solberg et al 2008**  **Model 2** | | Acute stroke patients >60 yrs., NOR (Hospital based) (Jan 1993- Nov 1999) | 1 year mortality | *missing /*734  (ND; *missing/*440 NV; *missing/*294) | logistic | 4 | 0.72 | 0.66 | 0.78 | 0.772 | | Internal (split sample) | | original model | |  |  |  |
| **Muscari et al 2011** **(BOAS)** | | Ischaemic stroke patients admitted to stroke unit, ITA (Hospital based (Feb 2007 -Feb 2008) | dead or mRS > 2 at 9 months | 129/221 | logistic | 5 | 0.85 | 0.77 | 0.92 | Calibration plot | | Internal (split sample) | | original model and scoring system | |  |  |  |
| **Sato et al 2014**  **Model 1** | | Minor ischemic stroke (Stroke unit multicentre observational study (SUMO)), JPN (Hospital based-84 centres) (Dec 2004- Dec 2005) | mRS 3–5 or death at 3 months | 203/1313 | Logistic | 9 | - | - | - | Calibration plot | | external(n=1) | | original model | |  |  |  |
| **Sato et al 2014**  **Model 2** | | Minor ischemic stroke (Stroke unit multicentre observational study (SUMO)), JPN (Hospital based-84 centres) (Dec 2004- Dec 2005) | mRS 3–5 or death at 3 months | 203/1313 | Logistic | 10 including baseline NIHSS | 0.67 | 0.63 | 0.70 | Calibration plot | | external(n=1) | | original model | |  |  |  |
| **Fullerton et al 1988** | | Acute stroke patients admitted to hospital , GB (Hospital based) (*missing)* | Recovered/ Independent/ Dependent/Dead at 6 months | 205 | logistic | 6 | - | - | - | - | | Internal: none External | | Original model | |  |  |  |
| **Oczkowski et al 1997** | | (Hospital based) (Oct 1990- Mar 1994) | FIM >/< 96 at discharge | /147 | neural network Analysis |  | - | - | - | - | | Internal  External (does not meet IC, N=17) | | - | |  |  |  |
| **Note: Only outcomes relevant to this review are listed, models may predict other outcomes also** ***ROC curves presented only, figures estimated from curve; ** 95% CI not stated, estimated using AUC and sample size; ***Calibration plot provided only**  **ND Number in derivation cohort; NV Number in validation cohort** | | | | | | | | | | | | | | | |  |  |  |
