# Supplementary material for: Clinical prediction models for mortality and functional outcome following ischemic stroke: A systematic review and meta-analysis
Source: PLoS One. 2018 Jan 29;13(1):e0185402. doi: 10.1371/journal.pone.0185402 (PMC5788336; doi:10.1371/journal.pone.0185402)
Supplement: S3 Table — (DOCX) [file pone.0185402.s007.docx]

S3 Table: Characteristics of included external validation studies

| Reference | Number of studies | Validation study | Population | n events/ Total N | Outcome | Predicted time point | Discrimination |  |  | Calibration  (p-value Hosmer-Lemeshow) | Recalibration |
| --- | --- | --- | --- | --- | --- | --- | --- | --- | --- | --- | --- |
|  |  |  |  |  |  |  | **AUC** | **LCL** | **UCL** |  |  |
| Ntaios et. al 2012  (ASTRAL) | 4 | Ntaois et al 2012 | Ischaemic stroke patients from Athens Stroke registry(ASR) (Jan 1998 - Dec 2010)  (Community based) | *missing*/1659 | mRs> 2 | 3 months | 0.94 | 0.91 | 0.97 | 0.22 | - |
|  |  |  | Ischaemic stroke patients from Vienna Stroke registry(VSR) ( Oct 1998- Dec 2001 )  (Community based) | *missing*/653 | mRs> 2 | 3 months | 0.77 | 0.72 | 0.82 | 0.49 | - |
|  |  | Liu et. al 2013 | Ischaemic stroke patients from Chinese National Stroke Registry  (Sep 2007- Aug 2008)  (Community based) | 1473/3715 | mRs> 2 | 3 months | 0.82 | 0.81 | 0.83 | - | - |
|  |  |  |  | 1349/ 3522 | mRs> 2 | 12 months | 0.81 | 0.80 | 0.83 | - | - |
|  |  | Ntaois et al. 2014 | Ischaemic stroke patients from ASTRAL  (Jan 2003- Mar 2011) (Hospital based registry data) | *missing*/1892 | mRs> 2 | 3 months | 0.85 | 0.762** | 0.802** | - | Ntaois et al. 2014 investigated the addition of information derived from multimodal modelling, but this did not increase the ASTRAL score's accuracy |
|  |  | Papavasileiou et al 2013 | Acute ischemic stroke patients from ASR (Jan 1998 - Dec 2010)  (Community based) | *missing/*1520 | mRs 3-6 | 5 years | 0.89 | 0.88 | 0.91 | - | - |
|  |  |  |  | *missing/*1521 | Mortality | 5 years | 0.81 | 0.78 | 0.83 | - | - |
| Counsell et. al 2002 (Six Simple variables) | 8 | Counsell et. al 2002 | Acute and sub acute stroke patients (exc. SAH) from SEPIVAC (1986-1989) and PCSS (1989-1990)  (Community based) | 87/538 | Alive | 30 days | 0.88 | 0.86 | 0.90 | *** | - |
|  |  |  |  | 33/538 | Alive and independent | 6 months | 0.84 | 0.82 | 0.86 | *** | - |
|  |  | Counsell et. al 2004 | Acute and subacute stroke patients (exc. SAH) from LHR(*Dates not stated*)  (Hospital based) | 90/1330 | Alive | 30 days | 0.87 | 0.86 | 0.89 | *** | - |
|  |  |  |  | 50/1330 | Alive and independent | 6 months | 0.84 | 0.83 | 0.85 | *** | - |
|  |  | Reid et. al 2007 | Hyper‐acute stroke patients  (2001-2002)  (Hospital based) | - | Alive | 30 days | - | - | - | - | - |
|  |  |  |  | 197/468 (IS only) | Alive and independent (mRs>/= 2) | 6 months | 0.779 (IS only) | 0.726** | 0.832** | *** | - |
|  |  | Counsell et. al 2003 | FOOD RCT  (Nov 1996 - Feb 2001)  (Hospital based (112 hospitals in 16 countries)) | 286/2955 | Alive | 30 days | 0.79 | 0.767** | 0.813** | *** | - |
|  |  |  |  | 1467/2955 | Alive and independent (mRs3-5) | 6 months | 0.78 | 0.758** | 0.802** | *** | - |
|  |  | Teale et al 2013 | Acute stroke patients from the prospective CLAHRC Cohort Study | 77/176 | Alive and independent (SIPSO score physical domain) | 6 months | 0.77 | 0.71 | 0.82 | *** | - |
|  |  |  |  | 77/176 | Alive and independent (OHS ≤2) | 6 months | 0.77 | 0.71 | 0.83 | *** | - |
|  |  | Lewis et al 2008 | Hyper acute ischaemic patients from Third International Stroke Trial trial of recombinant tissue plasminogen activator in ischaemic stroke (IST3- rt-PA) assessed within 6 hrs of symptom onset  (missing -Jan 2007) | 422/537 | Alive at | 30 days | 0.73 | 0.679** | 0.78 | *** | - |
|  |  |  |  | 179/537 | Alive and independent | 6 months | 0.78 | 0.68 | 0.82 | *** | - |
|  |  | Li et al 2012 | Chinese acute stroke patients in retrospective and prospective cohort study (Hospital based) (Oct 2007 - Mar 2009) | 107/248 | Alive | 30 days | 0.966 (IS only) | 0.94 | 1.00 | - | - |
|  |  |  |  | 52/248 | Alive and author defined independent | 6 months | 0.894 (IS only) | 0.85 | 0.97 | - | - |
|  |  | Thompson et al 2014 | prospective cohort study GBR (2002 - 2005) | 328/931 | Alive and independent (OHS≥ 3…higher OHS indicates poorer outcome?0=full recovery 6=dead) | 6 months | 0.72 | 0.70 | 0.74 | <0 | - |
| Seiffge et al 2014 (Six Simple variables-IVT) | 1 | Seiffge et al 2014 | Hyper acute stroke patients receiving intravenous thrombolysis (IVT) from observational IVT-databank study, CHE (June 1998–June 2012) | 136/638 | mRs 5-6 | 3 months | 0.786 | 0.74 | 0.83 | - | The adaptation of the SVM resulted in the exclusion of the variable ‘living alone before stroke’ and subsequently altered coefficients |
| Saposnik et. al 2011  (IScore) | 4 | Saposnik et. al 2011 | Ischaemic stroke patients from RCSNOSA (2002-2005)  (Community based (n=154 healthcare settings) | 380/3720 | mortality | 30 days | 0.79 | 0.770** | 0.810** | <0.001 | Recalibrated the regression model in the first half of the external validation sample and obtained predictions in the second half |
|  |  |  |  | 798/3720 | mortality | 1 year | 0.78 | 0.762** | 0.802** | <0.001 | Recalibrated the regression model in the first half of the external validation sample and obtained predictions in the second half |
|  |  | Saposnik et. al 2011 (b) | Ischaemic stroke patients from RCSN  (Jul 2003- Jun 2008) (Community based registry) | 2126 /3818 | Mortality or disability (MRS 3-5) | 30 days | 0.79 | 0.767** | 0.870** | 0.276 | - |
|  |  |  |  | 647 /3818 | Mortality or institutionalization | 30 days | 0.83 | 0.810** | 0.850** | 0.276 | - |
|  |  |  | Acute Ischaemic stroke patients from RCSNOSA  (Jul 2002- Jun 2009) (Hospital based registry) | 2043 /4365 | Mortality or disability (MRS 3-5) | 30 days | 0.68 | 0.662** | 0.696** | <0.001 | - |
|  |  |  |  | 751 /4365 | Mortality or institutionalization | 30 days | 0.74 | 0.725** | 0.761** | <0.001 | - |
|  |  | Bejot et. al 2013 | Acute ischaemic stroke patients from DSR  (Jan 2006 - Dec 2011) (Population based registry data) | 95/1092 | mortality | 30 days | 0.85 | 0.82 | 0.89 | 0.82 | - |
|  |  |  |  | 244/1092 | mortality | 1 year | 0.84 | 0.81 | 0.87 | 0.96 | - |
|  |  |  |  | 467/1092 | mRs 3-6 | Hospital discharge | 0.81 | 0.79 | 0.84 | 0.45 | - |
|  |  | Park et al 2013 | Acute ischemic stroke patients from Korean nationwide multicenter prospective observational study (Jan 2011- *missing)* (Hospital based) | 294/4061 | Mortality | 3 months | 0.86 | 0.84 | 0.88 | - | - |
|  |  |  |  | 1496/4061 | mRs 3 to 6 | 3 months | 0.82 | 0.81 | 0.83 | - | - |
| Flint et al 2010 (THRIVE) |  | Flint et al 2013 | Ischemic stroke patients form TREVO-2 RCT  (Hospital based)  (date missing) | Missing/ 178 | mRs 3-6 | 3 months | 0.719 | 0.631** | 0.807** | - | - |
| Kent et al 2006 (TPI) | 2 | McMeekin et al 2012 | Ischemic stroke patients treated with rt-PA from prospective SITS-ISTS, GBR (Dec 2002- Feb 2010) (Hospital based) | 1860/4022 stated bit this makes no sense? | mRS ≤1 | 3 months | 0.75 | 0.73 | 0.77 | *** | Tested whether 3 additional patient characteristics (congestive heart failure, signs of current infarction on pretreatment brain scan, and serum glucose5) would improve the explanatory power of the model for mRS ≤1 at 3 months |
|  |  |  |  | 2212/? | mRS ≥5 | 3 months | 0.79 | 0.77 | 0.81 | *** |  |
|  |  | Uyttenboogaart et al 2008 | Ischemic stroke patients treated with rt-PA from prospective registry University Medical Center Groningen, NLD (*missing* 2002 -Oct 2006) (Hospital based) | 104/301 | mRs 0-1 (4.5-hrs time window) | 3 months | 0.80 | 0.73** | 0.87** | - |  |
|  |  |  |  | 64/188 | mRs 0-1 (3-hrs time window) | 3 months | 0.82 | 0.73** | 0.91** | - |  |
|  |  |  |  | 40/113 | mRs 0-1 (3-4.5 hrs time window) | 3 months | 0.77 | 0.66** | 0.88** | - |  |
|  |  |  |  | 77/301 | mRs 5-6 (4.5-hrs time window) | 3 months | 0.78 | 0.71** | 0.85** | - |  |
|  |  |  |  | 50/188 | mRs 5-6 (3-hrs time window) | 3 months | 0.80 | 0.71** | 0.89** | - |  |
|  |  |  |  | 27/113 | mRs 5-6 (3-4.5 hrs time window) | 3 months | 0.74 | 0.61** | 0.85** | - |  |
| Kent et al 2015 (simplified- TPI) | 1 | Kent et al 2015 | Ischemic stroke patients treated with rt-PA from ECASS III doubleblind, parallel-group TRIAL  (Hospital based 130 sites in 19 European countries) (*date not stated)* | 299/778 | mRS ≤1 (using full NIHSS) | 3 months | 0.76 | 0.72 | 0.80 | *** | Simplified the original Stroke-TPI(Kent et al 2006) by reducing variables and interaction terms and by exploring simpler stroke severity scores |
|  |  |  |  | 299/778 | mRS ≤1(using 8-item score) | 3 months | 0.75 | 0.71 | 0.80 | *** |  |
|  |  |  |  | 299/778 | mRS ≤1(using 3-item score) | 3 months | 0.75 | 0.71 | 0.80 | *** |  |
|  |  |  |  | 389/778 | mRS ≤2(using full NIHSS) | 3 months | 0.80 | 0.74 | 0.86 | *** |  |
|  |  |  |  | 389/778 | mRS ≤2(using 8-item score) | 3 months | 0.79 | 0.73 | 0.85 | *** |  |
|  |  |  |  | 389/778 | mRS ≤2(using 3-item score) | 3 months | 0.79 | 0.73 | 0.85 | *** |  |
| Strbian et al 2012 (Dragon) |  | Strbian et al 2012 | University Hospital Basel, Basel, Switzerland (Hospital based) (*missing* 2005 and Jan 2011) | 189/333 | mRs 0-2 | 3 months | 0.80 | 0.74 | 0.86 | - | - |
|  |  |  |  | 78/333 | mRS 3– 4 | 3 months |  |  |  |  |  |
|  |  |  |  | 67/333 | mRs 5-6 | 3 months |  |  |  |  |  |
|  |  | Strbian et al 2013 | Acute ischemic stroke patients revieving IVT from a prospective database of 12 European and Australian stroke centers (Hospital based) *date missing* | 4519 | mRs 5-6 | 3 months | 0.84 | 0.82 | 0.85 | 0.94 | - |
|  |  |  |  | 4519 | mRs 0-2 | 3 months | 0.82 | 0.80 | 0.83 | 0.94 | - |
|  |  | Zang et al 2015 | Patients with acute ischemic stroke treated with t-PA on the TIMS-CHN prospective register (Hospital based(67 centers) (May 2007- April 2012) | 563/970 | mRs 0-2 | 3 months | 0.73 | 0.70 | 0.79 | - | - |
|  |  |  |  | 134/970 | mRs 5-6 | 3 months | 0.75 | 0.70 | 0.76 | - | - |
|  |  | Seiffge et al 2014 | Ischaemic stroke patients treated with IV-tPA from Helsinki Stroke Thrombolysis Registry, Finland (1995–2010) (Hospital based) | 180/1346 | mRs 5-6 | 3 months | 0.82 | 0.79 | 0.85 | - | - |
|  |  |  | Ischaemic stroke patients treated with IV-tPA from IVT databank, Basel, Switzerland  (June 1998–June 2012) (Hospital based) | 136/638 | mRs 5-6 | 3 months | 0.81 | 0.77 | 0.85 | - | - |
| Turc et al 2013 (MRI- DRAGON) |  | Turc et al 2014 | Ischaemic stroke patients treated with IV-tPA from Lille University Hospital registry, FRA (Hospital based) (May 2009 -Aug 2013) | 78/230 | mRs >2 | 3 months | 0.81 | 0.75 | 0.87 | 0.54 | - |
| Ringleb at al 2004  (ESRS) | 6 | Meng et al 2011 | Ischemic stroke patients from prospective CNSR, CHN (Sept 2007 - Aug 2008) (Hospital based) | 855/10323 (Ischemic stroke only) | Combined: Recurrent stroke, myocardial infarction, or cardiovascular mortality | 1 year | 0.60 | 0.59 | 0.62 | - | - |
|  |  | Weimar et al 2010 | Acute Ischemic stroke patients from prospective cohort study, DEU (Hospital based (N=10 centres) (Aug 2005 - Dec 2006) | 614/1607 (Ischemic stroke only) | Combined: Recurrent stroke or cardiovascular mortality | 1 year | 0.65 | 0.60 | 0.69 | - | - |
|  |  | Weimar et al 2009 | Ischemic stroke patients from the prospective REACH Registry (Hospital based (5473 sites in 44 countries) (Dec 2003 - June 2004) | 944/15605 | Combined: nonfatal stroke, nonfatal myocardial infarction, and cardiovascular mortality | 1 year | 0.60 | 0.58 | 0.62 | - | - |
|  |  | Andersen et al 2015 | Ischemic stroke patients from nationwide cohort study, NLD (Hospital based) (Jan 2003 - Dec 2012) | 3894/42 182 (mortality only) | Combined: stroke recurrence, mortality, and cardiovascular events (stroke, transient ischemic attack, myocardial infarction, or arterial thromboembolism) | 1 year | 0.65(mortality only) | 0.64 | 0.66 | - | - |
|  |  |  |  | 8942/42 182 (mortality only) | Combined: stroke recurrence, mortality, and cardiovascular events (stroke, transient ischemic attack, myocardial infarction, or arterial thromboembolism) | 5 years | 0.66 (mortality only) | 0.66 | 0.67 | - | - |
|  |  | Weimar et al 2008 | SCALA Cohort Study, DEU (July 2005 - Oct 2005) | 52/604 | Combined: recurrent cerebrovascular events, functional outcome or mortality |  |  |  |  | - | - |
|  |  | Maier et al 2013 | Ischaemic stroke patients who were admitted to the stroke unit between at the Klinikum Emden, DEU (2007 - 2011) (Hospital based) | 40/1727 | Mortality | 7 days | 0.58 | 0.49 | 0.66 | - |  |
|  |  |  |  | 30/1727 | mortality | in patient | 0.71 | 0.63 | 0.79 | - |  |
| Kernan et al 1991 SPI I | 1 | Kernan et al 2000 | Patients with carotid transient ischemia or minor stroke from (UK TIA(UK), CAPRIE and NoMaSS(USA) RCTS (Hospital based) (1979-1985;1992-1996;1990-1993 respectively) | 2662/9220 | Combined: stroke or mortality | 2 years | 0.59 | 0.57 | 0.60 | - | Yes, SPI II derived |
| Kernan et al 2000 SPI II | 4 | Meng et al 2011 | Ischemic stroke patients from prospective CNSR, CHN (Sept 2007 - Aug 2008) (Hospital based) | 1874/10323 (Ischemic stroke only) | Combined: Recurrent stroke, myocardial infarction, or cardiovascular mortality | 1 year | 0.61 | 0.59 | 0.62 | - | - |
|  |  | Weimar et al 2010 | Acute Ischemic stroke patients from prospective cohort study, DEU (Hospital based (N=10 centers) (Aug 2005 - Dec 2006) | 614/1607 (Ischemic stroke only) | Combined: Recurrent stroke or cardiovascular mortality | 1 year | 0.66 | 0.61 | 0.70 | - | - |
|  |  | Weimar et al 2013 | Ischaemic stroke patients undergoing in-patient rehabilitation from INSIGHT registry, DEU (May 2008–Sep 2008) (Hospital based(n=15 centers) | 93/856** | Combined: nonfatal stroke, nonfatal myocardial infarction, and cardiovascular mortality | 1 year | 0·60 | 0·57 | 0·64 |  | - |
|  |  | Kernan et al 2000 | Patients with carotid transient ischemia or minor stroke from RCTS (UK TIA(UK) & CAPRIE, NoMaSS(USA)  (Hospital based) (1979-1985,1992-1996,1990-1993) | 2572/9220 | Combined: stroke or mortality | 2 years | 0.63 | 0.62 | 0.65 | - | - |
| LeGall et al 1984 (SAPS I) | 1 | Handschu et al 2005 | Acute stroke patients in the NeuroCriticalCareUnit (NCCU) (Hospital based) *(missing)* | 29/90 | mortality | 10 days | 0.67 | 0.55 | 0.80 | - | - |
|  |  |  |  | 11/90 (IS only) | mortality | 3 months | 0.75 | 0.65 | 0.86 | - | - |
|  |  |  |  | 13/90 (IS only) | mortality | 1 year | 0.77 | 0.67 | 0.88 | - | - |
| (SAPS II) | 1 | Handschu et al 2005 | Acute stroke patients in the NeuroCriticalCareUnit (NCCU) (Hospital based) *(missing)* | 29/90 | mortality | 10 days | 0.68 | 0.57 | 0.80 | - | - |
|  |  |  |  | 11/90 (IS only) | mortality | 3 months | 0.77 | 0.67 | 0.97 | - | - |
|  |  |  |  | 13/90(IS only) | mortality | 1 year | 0.77 | 0.66 | 0.88 | - | - |
| Myint et al 2014  (SOAR) | 1 | Kwok et al 2013 | Stroke patients from Anglia Stroke and Heart Clinical Network , GBR (Hospital based(n=8)) (Sept 2008 - April 2011) | 303**/3547 | mortality | 7 days | 0.82 | 0.79 | 0.84 | - | - |
|  |  |  |  | 551**/3547 | mortality | In-hospital | 0.8 | 0.78 | 0.82 | - | - |
| Sato et al 2014  Model 1 | 1 | Sato et al 2014 | Ischemic stroke patients from prospective NCVC stroke register (Hospital based)  (Jan 2006 -Dec 2012) | 68**/879 | mRS 3–5 or mortality | 3 months | - | - | - | - | - |
| Sato et al 2014  Model 2 | 1 | Sato et al 2014 | Ischemic stroke patients from prospective NCVC stroke register (Hospital based)  (Jan 2006 -Dec 2012) | 68**/879 | mRS 3–5 or mortality | 3 months | - | - | - | - | - |
| Van Wijk et al 2005 (LLACI) | 1 | Weimar et al 2010 | Acute Ischemic stroke patients from prospective cohort study, DEU (Hospital based (N=10 centres) (Aug 2005 - Dec 2006) | 614/1607 (Ischemic stroke only) | Combined: Recurrent stroke or cardiovascular mortality | 1 year | 0.65 | 0.61 | 0.70 | - | - |
| Smith et al 2010 (GWTG)  Model 1 | 1 | Zhang et al 2012 | Ischaemic stroke patients from CNSR  (Hospital based)  (Sept 2007 - Aug 2008) | 203/7015 | mortality | In hospital | 0.867 | 0.839 | 0.895 | 0.674 | - |
| Smith et al 2010 (GWTG)  Model 2 | 1 | Zhang et al 2012 | Ischaemic stroke patients from CNSR  (Hospital based)  (Sept 2007 - Aug 2008) | 203/7015 | mortality | In hospital | 0.735 | 0.701 | 0.770 | 0.674 | - |
| APACHE IV |  |  |  |  |  |  |  |  |  |  |  |
| Bray et al 2014  Model 1 | 1 | Bray et al 2014 | Ischemic stroke/ primary ICH patients from SLSR, UK (Population based) (*missing* 2005- *missing* 2012) | 225**/1470 | Mortality | 30 days | 0.86(IS only) | 0.82 | 0.89 | Calibration plot | - |
| Bray et al 2014  Model 2 | 1 | Bray et al 2014 | Ischemic stroke/ primary ICH patients from SLSR, UK (Population based) (*missing* 2005- *missing* 2012) | 225**/1470 | Mortality (NIHSS consciousness only) | 30 days | 0.85 | 0.81 | 0.88 | Calibration plot | - |
| Wang et al 2003 | 1 | Li et al 2012 | Chinese acute stroke patients in retrospective and prospective cohort study (Hospital based) (Oct 2007 - Mar 2009) | 52/240 | Mortality | 1 year | 0.894  (IS only) | 0.846 | 0.965 | <0.05 | - |
| Johnston et al 2000  Model 1 | 1 | Johnston et al 2003 | Ischemic stroke patients from placebo group of (NINDS) rt-PA trial, US  (Hospital based)  (Jan 1991- Oct 1994) | 39/199 | BI >95 | 3 months | 0.83 | 0.920** | 0.740 | <0.05- | - |
| Johnston et al 2000  Model 2 | 1 | Johnston et al 2003 | Ischemic stroke patients from placebo group of (NINDS) rt-PA trial, US  (Hospital based)  (Jan 1991- Oct 1994) | 31/199 | GOS =1 | 3 months | 0.81 | 0.898** | 0.722** | -<0.05 | - |
| Johnston et al 2000  Model 3 | 1 | Johnston et al 2003 | Ischemic stroke patients from placebo group of (NINDS) rt-PA trial, US  (Hospital based)  (Jan 1991- Oct 1994) | 20/199 | NIHSS ≤1 | 3 months | 0.85 | 0.759** | 0.941** | -<0.05 | - |
| Johnston et al 2000  Model 4 | 1 | Johnston et al 2003 | Ischemic stroke patients from placebo group of (NINDS) rt-PA trial, US  (Hospital based)  (Jan 1991- Oct 1994) | 46/199 | GOS >2 | 3 months | 0.87 | 0.778** | 0.962 ** | -<0.05 | - |
| Johnston et al 2000  Model 5 | 1 | Johnston et al 2003 | Ischemic stroke patients from placebo group of (NINDS) rt-PA trial, US  (Hospital based)  (Jan 1991- Oct 1994) | 42/199 | BI ≤60 or death | 3 months | 0.88 | 0.788** | 0.972** | <0.05 | - |
| Johnston et al 2000  Model 6 | 1 | Johnston et al 2003 | Ischemic stroke patients from placebo group of (NINDS) rt-PA trial, US  (Hospital based)  (Jan 1991- Oct 1994) | 24/199 | NIHSS ≥ 20 or death | 3 months | 0.75 | 0.665** | 0.835** | -<0.05 | - |
| Johnston et al 2007  Model 1 | 1 | Johnston et al 2007 | Acute Ischaemic stroke patients form ASAP study, USA  prospective hospital based  (May 2000 -Aug 2005) | 167/266 | BI ≥ 95 | 3 months | 0.813 | 0.736** | 0.809** | - | - |
| Johnston et al 2007  Model 2 | 1 | Johnston et al 2007 | Acute Ischaemic stroke patients form ASAP study, USA  prospective hospital based  (May 2000 -Aug 2005)ASAP | 148/266 | mRs ≤1 | 3 months | 0.799 | 0.723** | 0.875** | - | - |
| Johnston et al 2007  Model 3 | 1 | Johnston et al 2007 | ASA Acute Ischaemic stroke patients form ASAP study, USA  prospective hospital based  (May 2000 -Aug 2005)P | 105/266 | NIHSS ≤1 | 3 months | 0.813 | 0.736** | 0.890** | - | - |
| Johnston et al 2007  Model 4 | 1 | Johnston et al 2007 | ASAP Acute Ischaemic stroke patients form ASAP study, USA  prospective hospital based  (May 2000 -Aug 2005) | 31/266 | mRs ≥5 | 3 months | 0.832 | 0.754** | 0.910** | - | - |
| Johnston et al 2007  Model 5 | 1 | Johnston et al 2007 | ASAP Acute Ischaemic stroke patients form ASAP study, USA  prospective hospital based  (May 2000 -Aug 2005) | 59/266 | BI ≤60 or death | 3 months | 0.833 | 0.755** | 0.911** | - | - |
| Johnston et al 2007  Model 6 | 1 | Johnston et al 2007 | ASAP Acute Ischaemic stroke patients form ASAP study, USA  prospective hospital based  (May 2000 -Aug 2005) | 33/266 | NIHSS ≥ 15 or death | 3 months | 0.882 | 0.802** | 0.962** | - | - |
| Johnston et al 2007  Model 7 | 1 | Johnston et al 2007 | ASAP Acute Ischaemic stroke patients form ASAP study, USA  prospective hospital based  (May 2000 -Aug 2005) | 167/266 | BI ≥ 95 | 3 months | 0.791 | 0.715** | 0.867** | - | - |
| Johnston et al 2007  Mode 8 | 1 | Johnston et al 2007 | ASAP Acute Ischaemic stroke patients form ASAP study, USA  prospective hospital based  (May 2000 -Aug 2005) | 148/266 | mRs ≤1 | 3 months | 0.758 | 0.684** | 0.832** | - | - |
| Johnston et al 2007  Model 9 | 1 | Johnston et al 2007 | ASAP Acute Ischaemic stroke patients form ASAP study, USA  prospective hospital based  (May 2000 -Aug 2005) | 105/266 | NIHSS ≤1 | 3 months | 0.791 | 0.715** | 0.867** | - | - |
| Johnston et al 2007  Model 10 | 1 | Johnston et al 2007 | ASAP Acute Ischaemic stroke patients form ASAP study, USA  prospective hospital based  (May 2000 -Aug 2005) | 31/266 | mRs ≥5 | 3 months | 0.830 | 0.753** | 0.907** | - | - |
| Johnston et al 2007  Model 11 | 1 | Johnston et al 2007 | ASAP Acute Ischaemic stroke patients form ASAP study, USA  prospective hospital based  (May 2000 -Aug 2005) | 59/266 | BI ≤60 or death | 3 months | 0.827 | 0.750** | 0.904** | - | - |
| Johnston et al 2007  Model 12 | 1 | Johnston et al 2007 | ASAP Acute Ischaemic stroke patients form ASAP study, USA  prospective hospital based  (May 2000 -Aug 2005) | 33/266 | NIHSS ≥ 15 or death | 3 months | 0.867 | 0.788** | 0.946** | - | - |
| Reid et al 2010 Model 1 (FSV) | 1 | Reid et al 2014 | Stroke patients admitted to hospital, CAN  (Hospital based)  (missing 2001 – missing 2002) | 538 | mRs≤2 | 6 months | - | - | - | - | - |
| Reid et al 2010 Model 2  (FSV) | 1 | Reid et al 2014 | Stroke patients admitted to hospital, CAN  (Hospital based)  (missing 2001 – missing 2002) | 538 | mRs≤2 | 6 months | - | - | - | - | - |
| Reid et al 2010 Model 3  (FSV) | 1 | Reid et al 2014 | Stroke patients admitted to hospital, CAN  (Hospital based)  (missing 2001 – missing 2002) | 538 | mRs≤2 | 6 months | - | - | - | - | - |
| Weimar et al 2002  M1 | 1 | GSSC et al 2004 | Ischemic stroke patients admitted to neurology dept. (n=13), DEU  (Hospital based)  Feb 2001- Mar 2002 | 831/1470 vs 639/1470 | BI ≥95 vs BI <95 or death | 100 days | - | - | - | - | - |
| Weimar et al 2002  M2 | 1 | GSSC et al 2004 | Ischemic stroke patients admitted to neurology dept. (n=13), DEU  (Hospital based)  Feb 2001- Mar 2002 | 113/1470 | Mortality | 100 days | - | - | - | - | - |
| Thrive | 2 | Flint et al 2013 | Ischemic stroke patients form TREVO-2 RCT  (Hospital based)  (date missing) | Missing /178 | mRS 3-6 | 3 months | 0.712 | 0.624** | 0.800** | - | - |
|  |  | Flint et al 2013 (b) | Ischemic Stroke patients from VISTA repository  (Hospital based)  (date missing) | 5724 | mRS 3-6 | 3 months | 0.756 | 0.740** | 0.772** |  |  |
| Allen et al 1984 (Guy’s Score) | 1 | Muir et al 1996 | GB  (Hospital based) | - | - | 3 months | 0.76 | 0.72 | 0.80 | - | - |
| Fullerton | 1 | Gladman et al 1992 | Acute stroke patients admitted to hospital, GB  (Hospital based) | 102 | - | - | - | - | - | - | - |
| *ROC curves presented only, figures estimated from curve; ** 95% CI not stated, estimated using AUC and sample size; ***Calibration plot provided only | | | | | | | | | | | |
