# Supplementary material for: Clinical prediction models for mortality and functional outcome following ischemic stroke: A systematic review and meta-analysis
Source: PLoS One. 2018 Jan 29;13(1):e0185402. doi: 10.1371/journal.pone.0185402 (PMC5788336; doi:10.1371/journal.pone.0185402)
Supplement: S1 Fig — (DOCX) [file pone.0185402.s010.docx]

S1 Fig: Meta analyses of models with internal validation only for mortality in hospital/at discharge
